# Molecular Insights into the Specific Targeting of *c-MYC* G-Quadruplex by Thiazole Peptides

**DOI:** 10.3390/ijms25010623

**Published:** 2024-01-03

**Authors:** Sen Cao, Qian Su, Yong-Hao Chen, Meng-Lu Wang, Yi Xu, Li-Hui Wang, Yan-Hua Lu, Jian-Feng Li, Jun Liu, Xiao-Jing Hong, Hong-Yan Wang, Jun-Ping Liu, Zhi-Guo Wang

**Affiliations:** 1 Institute of Ageing Research, School of Basic Medical Sciences, Hangzhou Normal University, Hangzhou 311121, China; 2021111012043@stu.hznu.edu.cn (S.C.); 2022111026058@stu.hznu.edu.cn (Q.S.); wanglihui@hznu.edu.cn (L.-H.W.); 20120068@hznu.edu.cn (Y.-H.L.); lijianfeng@hznu.edu.cn (J.-F.L.); junliu262@hznu.edu.cn (J.L.); 20197008@hznu.edu.cn (X.-J.H.); 20193010@hznu.edu.cn (H.-Y.W.); 2 School of Basic Medical Sciences, Hangzhou Normal University, Hangzhou 311121, China; 2021211301278@stu.hznu.edu.cn (Y.-H.C.); 2019211301185@stu.hznu.edu.cn (M.-L.W.); 2019211301182@stu.hznu.edu.cn (Y.X.)

**Keywords:** oncogene promotor, *c-MYC*, G-quadruplex, thiazole peptide, molecular docking, molecular dynamics, MM/GBSA

## Abstract

Stabilization of a G-quadruplex (G4) in the promotor of the *c-MYC* proto-oncogene leads to inhibition of gene expression, and it thus represents a potentially attractive new strategy for cancer treatment. However, most G4 stabilizers show little selectivity among the many G4s present in the cellular complement of DNA and RNA. Intriguingly, a crescent-shaped cell-penetrating thiazole peptide, **TH3**, preferentially stabilizes the *c-MYC* G4 over other promotor G4s, but the mechanisms leading to this selective binding remain obscure. To investigate these mechanisms at the atomic level, we performed an in silico comparative investigation of the binding of **TH3** and its analogue **TH1** to the G4s from the promotors of *c-MYC*, *c-KIT1*, *c-KIT2*, and *BCL2*. Molecular docking and molecular dynamics simulations, combined with in-depth analyses of non-covalent interactions and bulk and per-nucleotide binding free energies, revealed that both **TH3** and **TH1** can induce the formation of a sandwich-like framework through stacking with both the top and bottom G-tetrads of the *c-MYC* G4 and the adjacent terminal capping nucleotides. This framework produces enhanced binding affinities for *c-MYC* G4 relative to other promotor G4s, with **TH3** exhibiting an outstanding binding priority. Van der Waals interactions were identified to be the key factor in complex formation in all cases. Collectively, our findings fully agree with available experimental data. Therefore, the identified mechanisms leading to specific binding of **TH3** towards *c-MYC* G4 provide valuable information to guide the development of new selective G4 stabilizers.

## 1. Introduction

G-quadruplexes (G4s) are higher-order structures formed by Guanine-rich DNA or RNA sequences. In silico analyses of the human genome have identified over 376,000 putative quadruplex sequences that harbor a specific consensus motif (G_≥3_N_1–7_G_≥3_N_1–7_G_≥3_N_1–7_G_≥3_) [1,2]. Similarly, extensive experimental evidence has demonstrated the existence of G4s in a variety of nucleic acid regions, including the telomeric regions of chromosomes, the promotor regions of proto-oncogenes, 5′- and 3′-UTRs of mRNAs, tRNA fragments, the telomerase RNA component (TERC), and telomeric repeat-containing RNA (TERRA) [1,3,4,5,6,7]. The four-stranded G4s form protrusions on the nucleic acid structures and play important roles in a series of key biological processes including DNA replication, transcription and translation, and telomere maintenance [8,9,10,11,12,13,14].

Stabilization of G4s formed in the promotor regions of proto-oncogenes, such as *c-MYC*, has attracted considerable interest as a promising anticancer strategy [13,15]. Human *c-MYC* oncogene is located on chromosome 8, it involves in regulating gene expressions of ~15% of all genes through binding on enhancer boxes (E-boxes) [16,17]. A 27-mer G4-forming sequence (5′-TGGGGAGGGTGGGGAGGGTGG-GGAAGG-3′) has been identified in the nuclease hypersensitive element III1 of the *c-MYC* promoter region. This sequence, regulating up to 90% of *c-MYC* transcription, exists in equilibrium between its double-helical or single-stranded active form and the transcriptionally inactive G4 form [18]. A stable G4 structure has been determined by using the four consecutive 3′ runs of guanines within the 27-mer sequence [18]. The G4 has four loop isomers with dual G-to-T substitutions occurring at (14, 23), (11, 23), (14, 20), and (11, 20) positions, respectively [19]. The major (14, 23) substituted loop isomer with the sequence of 5′-TGAGGGTGGGTAGGGTGGGTAA-3′ (*c-MYC*14/23) forms the same structure as that of the WT *c-MYC* G4 [18,19]. All of the loop isomers show the same parallel conformation and contribute to the silencing of *c-MYC* in vivo [19].

Small molecules that stabilize the *c-MYC* G4 and down-regulate the expression of the *c-MYC* oncogene have been shown to inhibit the proliferation of cancer cells [20,21]. The most effective *c-MYC* G4 stabilizers have been shown to be compounds capable of forming π–π stacking interactions with the exposed G-tetrad layers, including berberines [22], carbazoles [23], naphthopyrones [24], porphyrins [5], and quindoline molecules [25]. These stabilizers are not selective enough for the *c-MYC* G4, however, and this lack of specificity has hampered their development into clinical treatments. Novel molecules with higher binding specificity as well as bioavailability are urgently needed.

**Figure 1:**
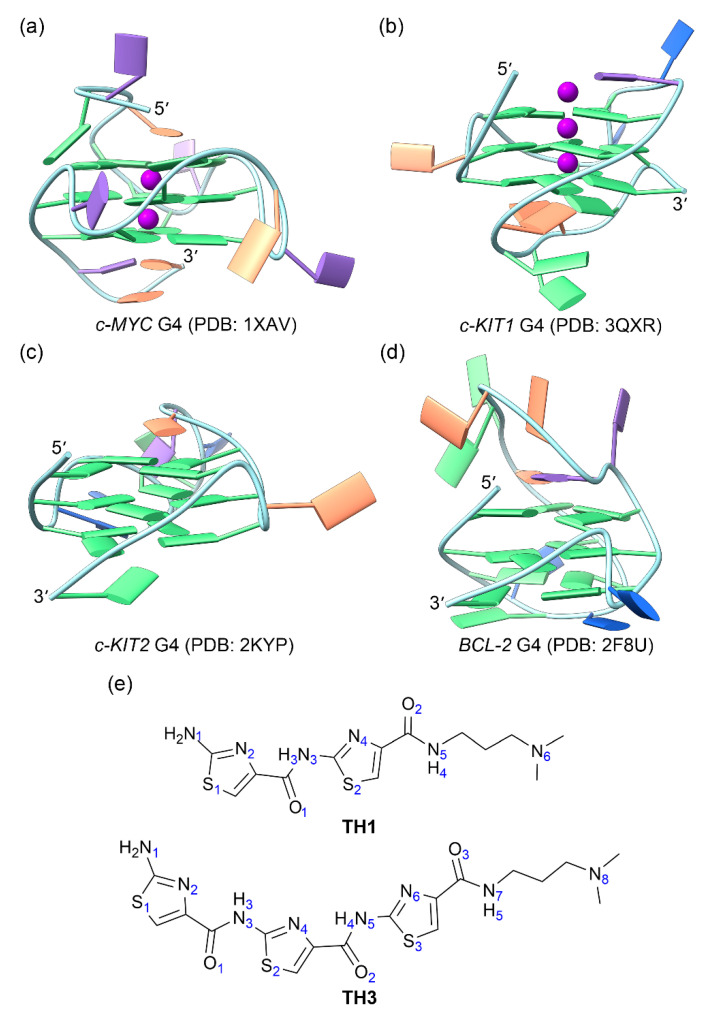
The structures of oncogene promotor G4s and the cell-penetrating thiazole peptides. (**a**) *c-MYC* G4; (**b**) *c-KIT1* G4; (**c**) *c-KIT2* G4; (**d**) *BCL2* G4; (**e**) peptides **TH1** and **TH3**. In G4 structures, the nucleotide bases of adenine (A), cytosine (C), guanine (G), and thymine (T) are colored orange, blue, green, and purple, respectively. The central potassium ions are represented by magenta spheres.

A series of crescent-shaped cell-penetrating thiazole peptides (**TH1**, **TH2**, and **TH3**) have been found to act as stabilizers of G4 structures. Intriguingly, one of these peptides, **TH3**, preferentially stabilizes the *c-MYC* G4 structure over other promotor G4s and thus specifically inhibits the expression of the *c-MYC* oncogene. **TH3** was found to exhibit antiproliferative activities by inducing S phase cell cycle arrest and apoptosis [26] (Figure 1, Table S1). However, the mechanism leading to the selective binding of thiazole peptides remains obscure.

In the current study, in order to gain atomic-level insight into this selective binding, molecular docking and explicit-solvent molecular dynamics (MD) simulations were performed on the binding of the *c-MYC* G4-selective peptide **TH3** and the less selective peptide **TH1** to multiple promotor G4 structures, including those associated with the promotors of *c-MYC*, *c-KIT1*, *c-KIT2*, and *BCL2*. We further characterized key binding features by performing combined analyses with information from principal component analysis (PCA), analyses of non-covalent interaction (NCI) and intermolecular hydrogen bonds, molecular mechanics/generalized Born surface area (MM/GBSA) calculations, and per-nucleotide binding free energy decompositions.

## 2. Results and Discussion

### 2.1. Dynamic Structural Feature of the Apo Promotor G4s

**Figure 2:**
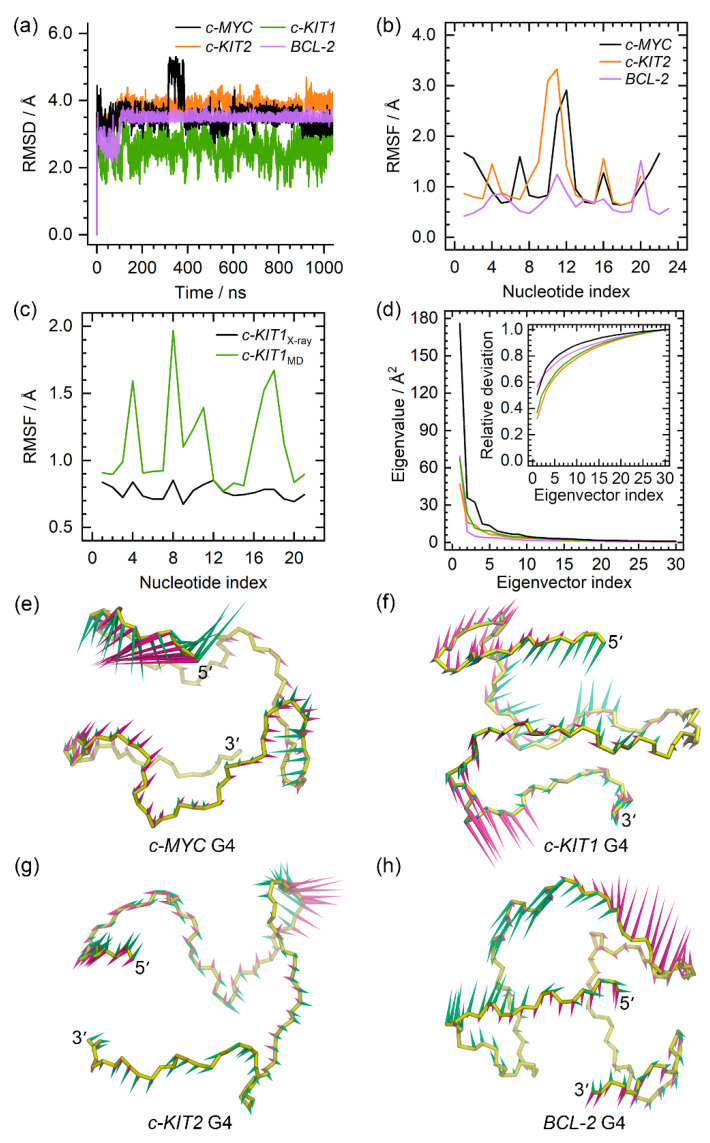
The dynamic features of the apo promotor G4s. (**a**) The root mean square deviation (RMSD) profiles of the apo G4s; (**b**) the root mean square fluctuation (RMSF) profiles of the *c-MYC*, *c-KIT2*, and *BCL2* G4s; (**c**) comparison of the RMSF profiles of the *c-KIT1* G4 derived from X-ray experiment and MD simulation; (**d**) the eigenvalue profiles constructed by the first 30 eigenvectors of G4s, with the profile of the *c-MYC*, *c-KIT1*, *c-KIT2*, and *BCL2* G4 colored black, green, orange, and purple, respectively; (**e**–**h**) porcupine plots of the dominant motions along the first (magenta) and the second (green) eigenvectors of the promotor G4s. The direction and size of the arrows represent the directions and extents of the principal motions of G4 backbone atoms along the corresponding eigenvector.

**Figure 3:**
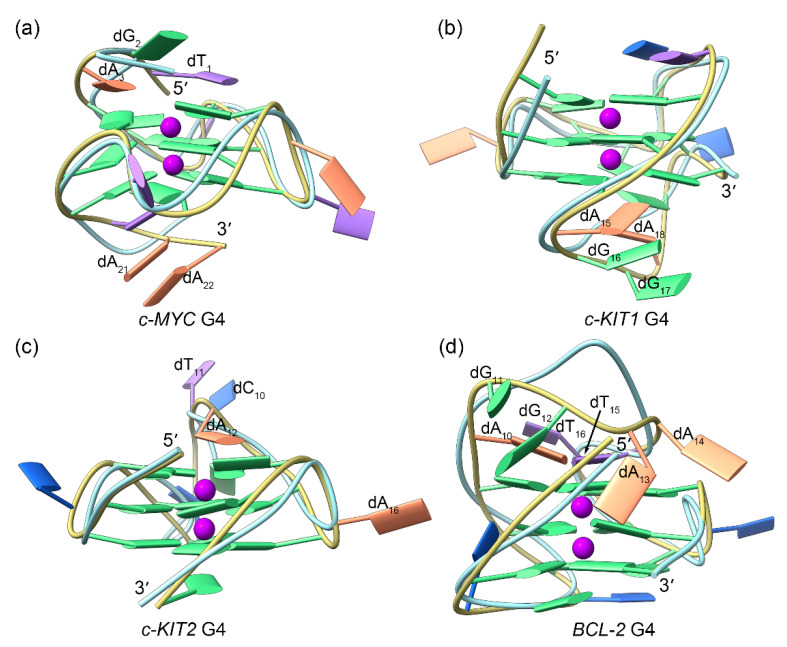
Conformational comparison between the MD-equilibrated and the solution NMR/X-ray structures of the promotor G4s, with the ribbons colored khaki and light blue, respectively. The central potassium ions are represented by magenta spheres.

The dynamic structural features of the G4s from the promotor regions of *c-MYC*, *c-KIT1*, *c-KIT2*, and *BCL2* were investigated in the absence of any binding factors. The features identified from MD simulations are summarized in Figure 2. The converged root mean square deviation (RMSD) fluctuations of G4s indicated that equilibrium states were achieved (Figure 2a). Conformationally flexible nucleotides were identified both by root mean square fluctuation (RMSF) analysis and by superimposing the equilibrated structures from MD simulations over the NMR or X-ray determined structures. In all of the G4 structures, the most flexible nucleotides were found to be located in the loop domains, as regional conformational variations were observed relative to original experimental structures (Figure 2b,c and Figure 3). Specifically in the c-MYC G4 structure, flexible nucleotides were also observed in the terminal domains (Figure 2b and Figure 3a). As expected, all of the guanine nucleotides that form the G-tetrad layers exhibited minimal conformational flexibility. In addition, our calculated RMSF profile for the *c-KIT* G4 exhibited similar fluctuations to those determined experimentally, providing further validation of the current simulations.

PCA revealed that 42.99% to 63.13% of essential motions can be represented by the first two eigenvectors of promotor G4s (Figure 2d). Porcupine plots revealed that the first two eigenvectors mainly corresponded to the motions of the 5′-termini of *c-MYC* G4, the three loops of *c-KIT1* G4, and the second loops of the *c-KIT2* and *BCL2* G4s (Figure 2e–h). The identified dynamic features of all G4s were consistent with the RMSF profiles and the conformational comparisons.

### 2.2. Docking-Derived Binding Mode of Promotor G4s to the Peptides

**Figure 4:**
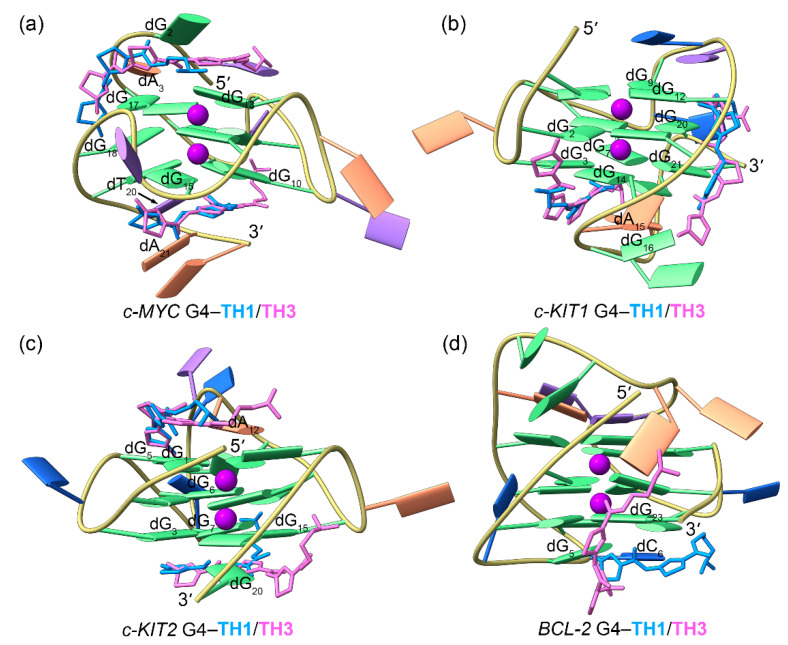
Binding modes of **TH1**/**TH3** and MD-equilibrated promotor G4s derived from molecular docking calculations. **TH1** and **TH3** are colored royal blue and orchid, respectively. The central potassium ions are represented by magenta spheres.

Thiazole peptides **TH1** and **TH3** under the neutral state were individually docked to the MD-equilibrated structures of G4s. As shown in Figure 4, in *c-MYC*, *c-KIT1*, and *c-KIT2* G4s, two binding sites were predicted for both **TH1** and **TH3**, with both peptides mainly stacking to the exposed top and bottom G-tetrads of *c-MYC* and *c-KIT2* G4s while intercalating into the minor grooves of the *c-KIT1* G4 (Figure 4a–c). Notably, the binding conformations of both peptides superimposed well in these three G4s. Conversely, due to the steric hindrance generated by the second loop to the top G-tetrad, only one molecule of a peptide can bind to the *BCL2* G4, specifically at the bottom G-tetrad; in this case, **TH1** and **TH3** exhibited different orientations (Figure 4d). Detailed information describing the interactions between G4s and the binding peptides, including intermolecular hydrogen bonds, π-π stacking interactions, and docking evaluated binding affinities, are summarized in Table 1. **TH1** and **TH3** under the protonated state exhibited similar binding mode to the neutral ones (Figure S1) but showed generally decreased intermolecular hydrogen bond interactions with lower binding affinity (Table S2). Therefore, more attention was paid to the analysis of neutral thiazole peptides.

In all four cases, **TH3** was calculated to have a stronger binding affinity than **TH1** (Table 1); this finding is in agreement with the more potent antiproliferative activity of **TH3**. However, the affinity evaluation did not take into account the induced fit of the peptides when interacting with G4s. In addition, these evaluations do not deal with solvation effects precisely. Accordingly, *c-KIT1* G4 was inaccurately predicted as the preferred binding target for these *c-MYC*-specific peptides. Therefore, we concluded that MD simulations are necessary for correctly identifying the binding feature of the G4–peptide complexes and further evaluating the corresponding binding affinities.

### 2.3. Dynamic Features of the G4–TH1/TH3 Binding Complexes

**Figure 5:**
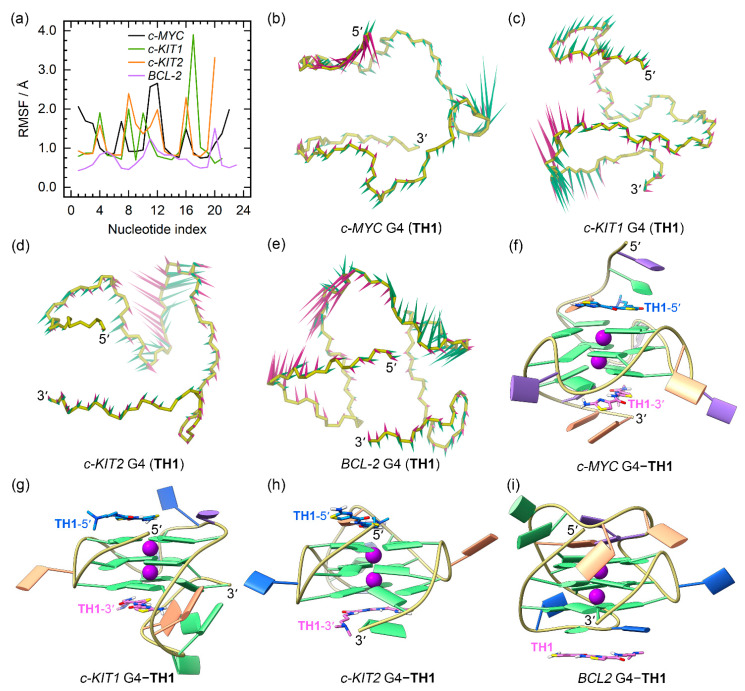
The dynamic features of promotor G4−**TH1** binding complexes. (**a**) The RMSF profiles of **TH1** bound G4s; (**b**–**e**) porcupine plots of the dominant motions along the first (magenta) and the second (green) eigenvectors of **TH1** bound G4s; (**f**–**i**) the equilibrated conformations of promotor G4–**TH1** binding complexes. **TH1**-5′ and **TH1**-3′ indicate the corresponding **TH1** peptide stacks to the top and bottom G-tetrad, with the carbon atoms colored royal blue and orchid, respectively. The central potassium ions are represented by magenta spheres.

The RMSD profiles of all the G4–**TH1** binding complexes converged at the final MD stages (the last 300 ns), indicating that they reached their equilibrium (Figure S2a−d). The RMSF profiles of the **TH1**-bound G4s exhibited similar patterns to those observed for apo G4s. However, the 5′- and 3′-termini of the *c-MYC* G4 and the 3′-termini of the *c-KIT2* G4 showed elevated flexibility when bound to **TH1**, indicating conformational variations induced by **TH1** binding (Figure 5a).

PCA demonstrated that 30.09% to 69.11% of the essential motions of promotor G4s can be represented by the first two eigenvectors (Figure S2e). Porcupine plots illustrated a correspondence of most of the motions to conformational fluctuations of the 5′-termini and the second loop of the *c-MYC* G4, the third loop of the *c-KIT1* G4, and the second loops of *c-KIT2* and *BCL2* G4s (Figure 5b−e). The equilibrated structures of G4–**TH1** complexes showed significant discrepancies in the **TH1** binding conformations relative to the conformations identified in docking-derived structures. As shown in Figure 5f–i, both **TH1** molecules formed better stacking framework with *c-MYC* and *c-KIT2* G4s than that in the docking-derived binding conformations. Surprisingly, the initial intercalation binding form was altered to the end-stacking mode in the *c-KIT1* G4–**TH1** complex. In the *BCL2* G4–**TH1** complex, instead of binding to the bottom G-tetrad, **TH1** stacked to the first loop nucleotides, demonstrating that this complex also underwent a drastic change in the mechanism of binding.

**Figure 6:**
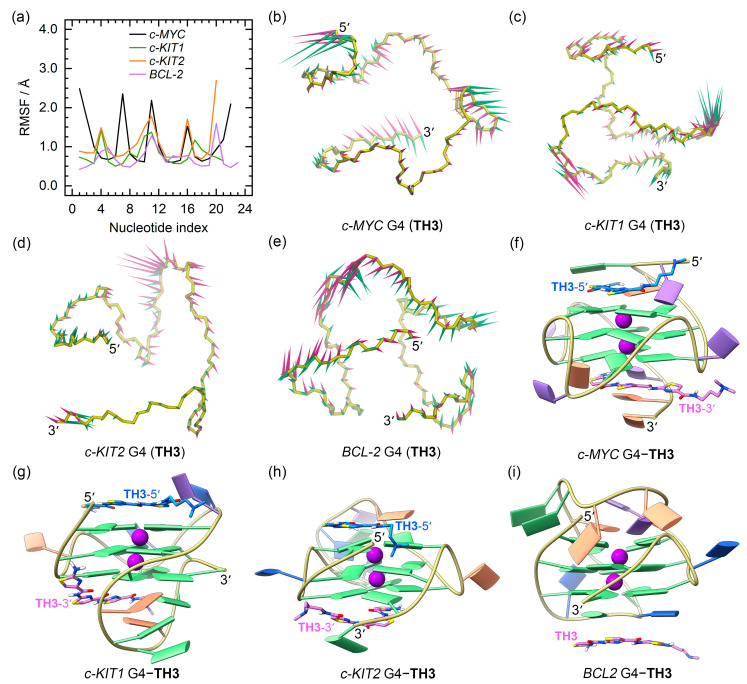
The dynamic features of promotor G4–**TH3** binding complexes. (**a**) The RMSF profiles of **TH3**-bound G4s; (**b**–**e**) porcupine plots of the dominant motions along the first (magenta) and the second (green) eigenvectors of **TH3**-bound G4s; (**f**–**i**) the equilibrated conformations of promotor G4–**TH3** binding complexes. **TH3**-5′ and **TH3**-3′ indicate the corresponding **TH3** peptide stacks to the top and bottom G-tetrad, with the carbon atoms colored royal blue and orchid, respectively. The central potassium ions are represented by magenta spheres.

For **TH3**-bound promotor G4s, we observed more rapid convergences of the RMSD profiles with decreased values compared to similar analyses of the apo and **TH1**-bound structures (Figure S3a–d). In addition, loop flexibilities were reduced as shown by the overall decreased RMSF profiles (Figure 6a), consistent with an increased stabilizing effect of **TH3**.

PCA demonstrated that 24.79% to 52.17% of the essential motions of promotor G4s can be represented by the first two eigenvectors (Figure S3e), which mainly corresponded to the essential motions of both the terminus and the second loop of the *c-MYC* G4, the second and the third loops of the *c-KIT1* G4, and the second loops of the *c-KIT2* and *BCL2* G4s, with the motion levels apparently decreased relative to **TH1**-bound G4s (Figure 6b–e). In the equilibrated structures, both **TH3** molecules were directly stacked to the exposed top and bottom G-tetrads of *c-MYC*, *c-KIT1*, and *c-KIT2* G4s, with a single **TH3** molecule stacking to the first loop nucleotides of the *BCL2* G4 (Figure 6f–i).

The above findings demonstrated that **TH1** and **TH3** share a similar mode of binding. The 2:1 binding stoichiometry of **TH3** and the *c-MYC* G4 and the end-stacking binding mode were consistent with the findings from fluorimetric titrations, NMR titrations, and the chemical shift perturbations analysis [26]. It should be noted that **TH1** and **TH3** exploited the conjugated thiazole groups to form π-π stacking interactions with G4 bases, with the terminal dimethylamino groups presenting outward orientations (Figure 5f–i and Figure 6f–i). This further suggests that the protonation state of the dimethylamino group would hardly create any significant impact on the binding mode of thiazole peptides.

### 2.4. Noncovalent Interactions Mediating the Binding of G4s to TH1 and TH3

**Figure 7:**
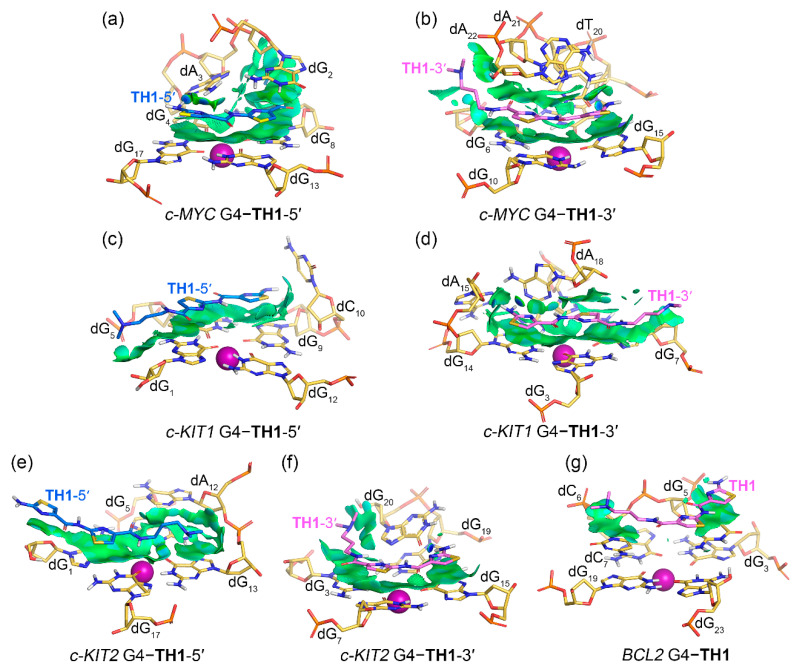
Noncovalent interactions in the MD-equilibrated promotor G4–**TH1** binding complexes shown as NCI surfaces (isovalue of 0.3 au). The nucleotides that involve in the noncovalent interactions are shown and labeled. The central potassium ions are represented by magenta spheres.

**Figure 8:**
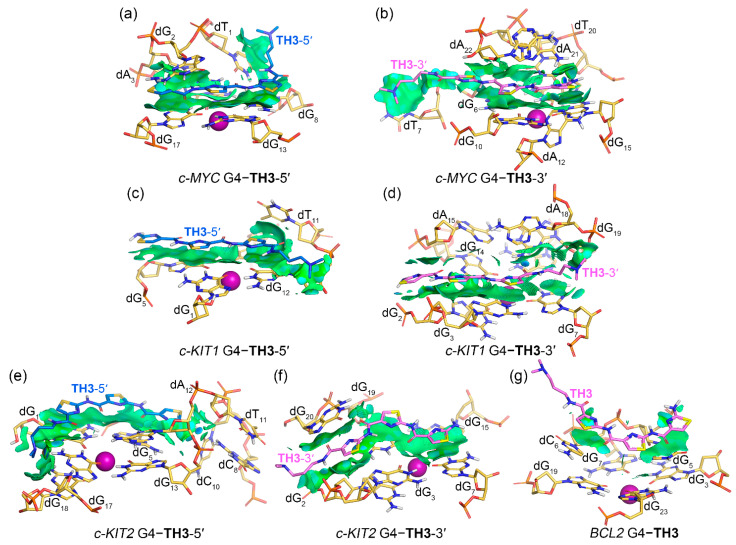
Noncovalent interactions in the MD-equilibrated promotor G4–**TH3** binding complexes shown as NCI surfaces (isovalue of 0.3 au). The nucleotides that involve in the noncovalent interactions are shown and labeled. The central potassium ions are represented by magenta spheres.

Noncovalent interactions between promotor G4s and peptides **TH1** and **TH3** were rendered as isosurfaces with an NCIplot (Figure 7 and Figure 8). Both **TH1** and **TH3** that stacked over the top G-tetrad of the *c-MYC* G4 formed extensive van der Waals (vdW) interactions with dG_8_, dG_13_, and dG_17_. **TH1** exhibited additional vdW interactions with dG_2_ and hydrogen bond interactions with dA_3_, while **TH3** was found to form additional vdW interactions with dT_1_ and dG_2_ and hydrogen bond interactions with dA_3_ (Figure 7a and Figure 8a). For both peptides that stacked below the bottom G-tetrad of the *c-MYC* G4, extensive vdW interactions with G-tetrad nucleotides dG_6_, dG_10_, and dG_15_ and 3′-terminal nucleotides dA_21_ and dA_22_ were found, with **TH3** showing additional vdW interactions with dT_7_. Hydrogen bond interactions of both peptides with dT_20_ are shown by blue isosurfaces in Figure 7b and Figure 8b. It should be noted that both top- and bottom-stacked **TH1** and **TH3** formed sandwich-like frameworks with the corresponding G-tetrad and terminal nucleotides, facilitating specific recognition and strong binding affinity.

In binding with *c-KIT1* and *c-KIT2* G4s, both peptides mainly stacked to the top and bottom G-tetrads via vdW interactions without forming sandwich frameworks, except for the bottom G-tetrad stacked **TH3** that formed additional vdW interactions with dG_20_ of *c-KIT2* G4 (Figure 7c–f and Figure 8c–f). Notably, the corresponding isosurfaces were more fragmented and less extensive compared to the isosurfaces observed in *c-MYC* G4–**TH1/TH3** complexes, indicating decreased binding specificity and affinity. For binding with *BCL2* G4, both **TH1** and **TH3** barely stacked to the first loop nucleotides, dG_5_ and dC_6_, via vdW interactions, with the two localized isosurfaces correlating their weaker binding affinities.

### 2.5. Intermolecular and Intramolecular Hydrogen Bonds

Intermolecular hydrogen bonds represent key contributions to biomolecular interactions. In the present context, we comprehensively searched for intermolecular hydrogen bonds using the criteria of bond length < 3.5 Å and bond angle > 120°. The typical requirement of hydrogen bond occupancy (HBO) > 30% was not used so as to capture cases in which the binding peptides formed stable hydrogen bonds with G4s during late stages of MD simulations. All the intermolecular hydrogen bonds are listed in Table 2, and the variations in distance between the hydrogen bond donor and acceptor atoms throughout the MD simulations are displayed in Figure S4.

Overall, both **TH1** and **TH3** were found to more frequently play the role of hydrogen bond donor, while G4 nucleotides more frequently played the role of hydrogen bond acceptor. When peptides formed sandwich-like complexes, such as **TH1** with *c-MYC* G4 and **TH3** with *c-MYC* and *c-KIT2* G4s, they showed greatly increased hydrogen bond interactions, both in the roles of hydrogen bond donors and receptors. This phenomenon was especially strong for the *c-MYC* G4–**TH3** binding complex, in which all of the intermolecular hydrogen bonds exhibited over 30% occupancies, indicative of the high binding stability. Notably, an occupancy of over 30% does not necessarily mean that a hydrogen bond existed in the equilibrated structure, as relative positional changes in the ligand and receptor during late stages of MD simulation may have led to a loss of such a bond. For example, this phenomenon became apparent in the analyses of the *BCL2* G4–**TH1** complex (Figure S4j). Therefore, distance analysis is essential to the accurate identification of hydrogen bond interactions.

**Figure 9:**
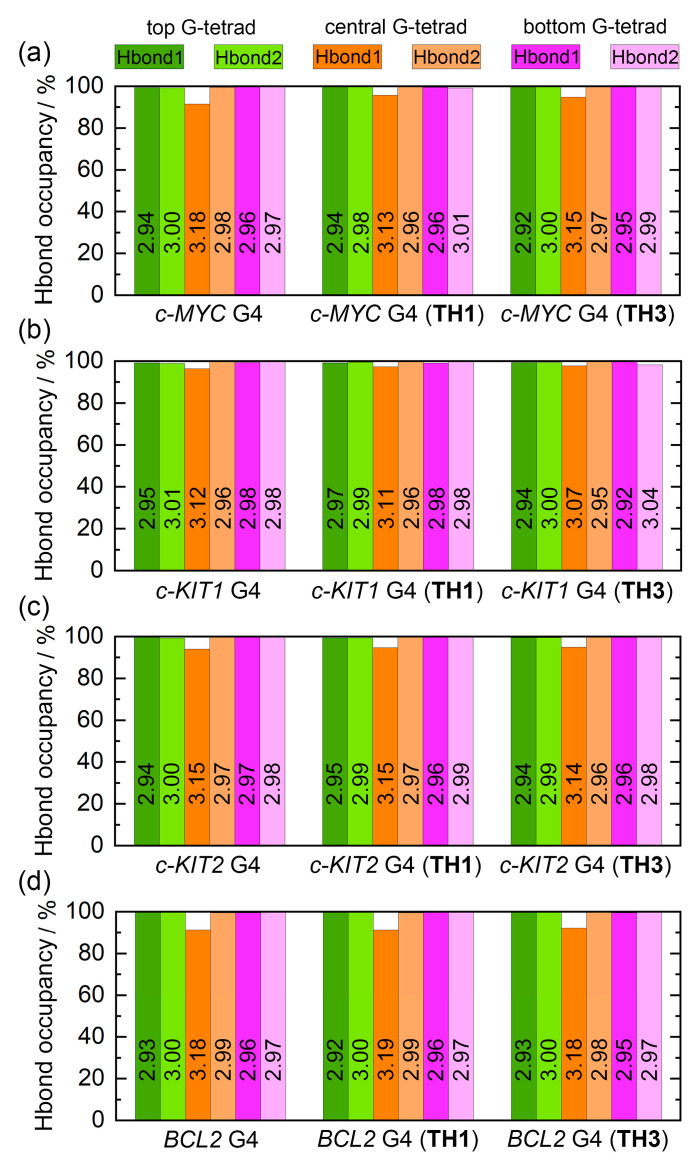
Hoogsteen hydrogen-bond analysis. (**a**–**d**) The hydrogen bonds of N1–H1···O6 and N2–H21···N7 are denoted as Hbond1 and Hbond2, respectively. Occupancy of hydrogen bonds in the top, central, and bottom G-tetrads are represented by differently colored columns. The G-tetrad layer-averaged hydrogen-bond lengths are labeled in the corresponding columns.

Intramolecular Hoogsteen hydrogen bonds that formed within the coplanar G-tetrad, including N1–H1···O6 (Hbond1) and N2–H21···N7 (Hbond2), were found to contribute strongly to the maintenance of the G4 structure. The state of these bonds thus may serve as a marker of G4 structural stability. The G-tetrad layer-averaged Hoogsteen HBOs and the average bond lengths for the apo and the peptide-bound G4s are summarized in Figure 9. All of the Hoogsteen hydrogen bonds were highly stable throughout MD simulations, as indicated by the HBOs and bond lengths (Table S3). Interestingly, all of the exposed top and bottom G-tetrads exhibited maximum HBOs, whereas Hbond1 of the central G-tetrad exhibited decreased HBOs. Upon peptide binding, a general increasing trend of HBO was uncovered, especially for Hbond1 of the central G-tetrad. Specifically, the minimum HBOs of 91.54%, 96.45%, 94.05%, and 91.33% increased to 94.89%, 97.39%, 94.68%, and 91.36% for *c-MYC*, *c-KIT1*, *c-KIT2*, and BCL2 G4s, respectively (Figure 9). Comparatively, both peptides showed stronger stabilizing effects toward *c-MYC* and *c-KIT1* G4s relative to the other promotor G4s.

### 2.6. Binding Free Energies between the Promotor G4s and TH1/TH3

With the ability to make good predictions on the hydration free energy for charged molecules when considering the relative solvation free energy, MM/GBSA calculations were performed to evaluate the binding affinities between promotor G4s and **TH1** or **TH3**. As summarized in Table 3, electrostatic interactions (∆*E*_ele_), vdW interactions (∆*E*_vdW_), and non-polar solvation effects (∆*G*_SA_) were all found to support the binding interactions, with vdW interactions contributing the most binding energy. In contrast, the contributions from polar solvation effects (∆*G*_GB_) and entropies (T∆S) were unfavorable. In binding with every promotor G4, under the same binding position, **TH3** consistently exhibited increased binding free energy relative to **TH1**, consistent with the observed superiority of **TH3** in terms of antiproliferative effects.

The overall binding free energies of **TH3** followed an order of *c-MYC* G4 (−71.2 kcal∙mol^−1^) ≫ *c-KIT1* G4 (−53.1 kcal∙mol^−1^) > *c-KIT2* G4 (−45.0 kcal∙mol^−1^) ≫ *BCL2* G4 (−7.1 kcal∙mol^−1^), supporting its high binding specificity for the *c-MYC* G4. In addition, the binding affinity order fully agrees with its stabilizing priorities accessed through FRET melting assays, providing validation of the power of MM/GBSA in ranking the relative binding affinities [26].

**TH1** showed a similar binding priority to promotor G4s, namely *c-MYC* G4 (−49.4 kcal∙mol^−1^) > *c-KIT1* G4 (−41.2 kcal∙mol^−1^) > *c-KIT2* G4 (−28.2 kcal∙mol^−1^) ≫ BCL2 G4 (−6.9 kcal∙mol^−1^). Comparatively, these results demonstrate that the binding affinities were lower for **TH1**; in addition, **TH3** exhibited more selective binding for the *c-MYC* G4 relative to the *c-KIT1* G4 than **TH1** (Table 3).

It is worth noting that the binding affinity between **TH3** and *BCL2* G4 appears to be underestimated, since the difference in melting temperatures (Δ*T*_m_) determined in FRET melting assays is much smaller than the affinity difference. Specifically, the Δ*T*_m_ value determined for the *c-KIT2* G4 was 7.6 °C, and the Δ*T*_m_ value determined for the *c-BCL2* G4 was 7.1 °C. In comparison, the Δ*T*_m_ value determined for the *c-MYC4* G4 was 22.0 °C. Similar findings also apply to **TH1**. Considering the loop-stacking binding mode identified in the *BCL2* G4, we propose that under in vitro experimental conditions, a second molecule of *BCL2* G4 may be recruited by the fully exposed **TH3** or **TH1**, resulting in the formation of a higher-order binding complex mediated by either peptide. This complex likely promotes the binding affinity to a level similar to that observed for complexes of the peptides with *c-KIT2* G4 [27].

**Figure 10:**
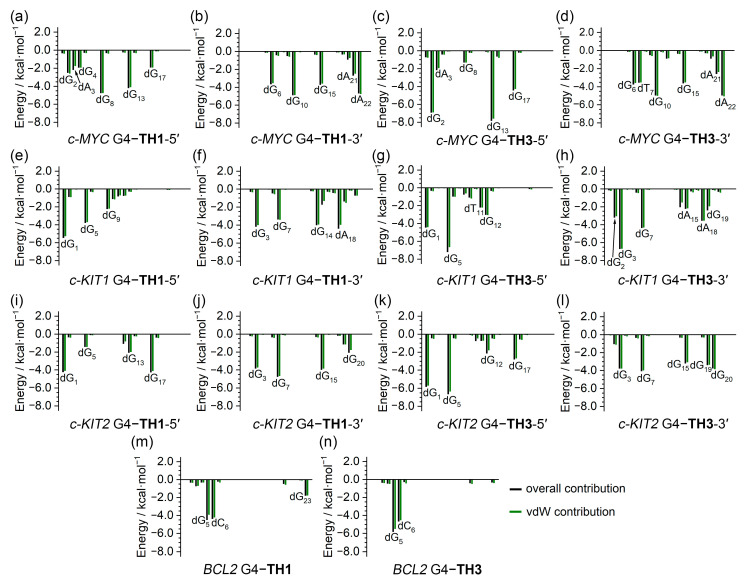
Per-nucleotide decomposition of the binding free energy. The contributions from vdW interactions (green) basically equal the combined overall contributions (black) from electrostatic interactions, vdW interactions, and solvation effects in every case.

The binding free energies were further investigated to determine per-nucleotide contributions, under conditions in which entropic contributions were excluded. Intriguingly, the per-nucleotide free energy contributions calculated based on the trajectories of the last 200 ns of MD simulations were consistent with the information shown in the NCIplot, which was based on MD-equilibrated structures. In addition, per-nucleotide vdW contributions were essentially equal to the overall contributions in all cases, indicating the pivotal role of vdW interactions in promoting the binding of G4s to thiazole peptides (Figure 10). It is noteworthy that the non-G-tetrad nucleotides that were involved in the sandwich-like binding frameworks, such as dG_2_ and dA_22_ of the *c-MYC* G4, made significant contributions to the binding, providing valuable support for the specific binding with the *c-MYC* G4.

## 3. Materials and Methods

### 3.1. Data

The solution NMR structures of *c-MYC*, *c-KIT2*, and *BCL2* G4s together with the X-ray structure of *c-KIT1* G4 were retrieved from the PDB data bank with the IDs of 1XAV [18], 2KYP [28], 2F8U [29], and 3QXR [30], respectively (Figure 1a–d). As the central potassium ions are necessary to G4 structure stability [31,32,33], the models with two K^+^ intercalated between the adjacent G-tetrads were generated for *c-KIT2* and *BCL2* G4s by using the UCSF ChimeraX software (version 1.6.1) [34]. To remain consistent with the experimental nucleotide sequence (Table S1), the structures were edited by removing redundant terminal nucleotides. The K^+^ located above the top G-tetrad in the *c-KIT1* G4 was removed due to its unstable binding [32]. The structures of **TH1** and **TH3** were built with the GaussView software (version 6.0.16) (Figure 1e) and were optimized through applying the density functional theory (DFT) at the level of B3LYP/6-31G(d). The atomic charges were further calculated for both compounds by using the restricted electrostatic potential (RESP) method with Gaussian 03 at the level of HF/6-31G(d).

### 3.2. Molecular Docking

Molecular docking calculations were performed by using the AutoDock Vina 1.2.5 software [35]. The receptors of G4s and the ligands of thiazole peptides were prepared with the AutoDockTools software (version 1.5.6) [36]. The Gasteiger charges were computed for both receptors and ligands, with the non-polar hydrogen atoms merged. All the rotatable bonds of **TH1** and **TH3** were set flexible, while the G4s were set rigid. In each docking calculation, a cubic box centered at the G4 geometric center comprising 90 × 90 × 90 grids with a grid spacing of 0.375 Å was used to define the possible binding region. The box was large enough to encompass every G4 structure so that no binding modes were excluded. All other parameters were set as the default. Five independent docking calculations with the exhaustiveness parameter of 25 were performed for each ligand in order to obtain the energetically favored and the most populated binding conformations [37]. In addition, as the terminal dimethylamino groups of **TH1** and **TH3** have a pKa value close to 10.0 [38] and their protonation state may change upon binding to promotor G4s [39], molecular docking calculations were performed with **TH1** and **TH3** under both neutral and protonated states.

### 3.3. Molecular Dynamics

Amber 22 software was used for MD simulations [40,41]. Each of the apo G4s and the docking-predicted binding complexes were placed at the center of a truncated octahedron box of TIP3P water molecules at a margin distance of 10.0 Å. Environmental K^+^ ions were added to maintain electrical neutrality. The previously validated FF99SB force fields with parmbsc1 and χ_OL3+OL15_ modifications were applied for G4 [42,43]. The calibrated parameter (radius 1.705 Å, well depth 0.1936829 kJ·mol^−1^) and the standard Amber parameter (radius 2.658 Å, well depth 0.00328 kJ·mol^−1^) were used for the central and environmental K^+^ ions, respectively [27]. For **TH1** and **TH3**, the second generation of general Amber force field (GAFF2) was applied [42]. Each model was first energy minimized for 10,000 steps by using the steepest descent minimization method with a harmonic constraint of 500 kcal mol^−1^ Å^−2^ imposed on the apo G4s or the complexes, followed by a conjugated gradient minimization for 10,000 steps with no constraint. Then, the system was gradually heated from 0 to 300 K under the NVT ensemble for 500 ps, with a weak constraint of 10 kcal mol^−1^ Å^−2^ imposed on the apo G4s or the complexes. The model was subsequently subjected to an equilibrium simulation for 1 ns by removing all constraints. Finally, the production simulation for each model was conducted under the NPT ensemble, with the simulation time ranging from 1000 ns to 1200 ns. In all MD simulations, parameters were set according to our previous reports [27]. MD trajectories were recorded at an interval of 10 ps for structural and energetic analyses.

### 3.4. Principal Component Analysis

PCA was performed to describe the essential motions of G4s by removing the overall translational and rotational movements from MD trajectories [44]. Based on 10,000 frames evenly extracted from the last 200 ns of MD trajectories, PCA of G4 backbones was carried out for each model using the CPPTRAJ module of AmberTools. The graphical summaries of essential motions along the first two eigenvectors were produced as porcupine plots using the VMD software (version 1.9.4) [45].

### 3.5. Noncovalent Interactions

NCIplot calculations were carried out with a step size of 0.10 to visualize the interacting regions between G4 and the binding peptides [46]. The reduced gradients were rendered as an isosurface in VMD, using an isovalue of 0.3 au.

### 3.6. Binding Free Energy Analysis

The binding free energies between G4 and the binding peptides were evaluated with MM/GBSA calculations. A total of 500 snapshots evenly extracted from the last 200 ns of the MD trajectory were used for the calculation of each binding complex. The binding free energy value is equal to the free energy difference between the binding complex (*G*_complex_) and the sum of receptor (*G*_rec_) and ligand (*G*_lig_) as follows:

∆*G*_bind_ = *G*_complex_ − (*G*_rec_ + *G*_lig_). (1)

Each item can be calculated with the following equation:

∆*G*_bind_ = ∆*H* − T∆*S* ≈ ∆*E*_MM_ + ∆*G*_solv_ − T∆*S,* (2)

where ∆E_MM_ is the molecular mechanical energy of the gas phase, ∆*G*_solv_ is the solvation free energy, and T∆*S* is the contribution of entropy. ∆E_MM_ comprises contributions from electrostatic energy (∆*E*_ele_), van der Waals (vdW) interaction energy (∆*E*_vdW_), and internal strain energy (∆*E*_int_) which includes bonds, angles, and dihedral energies that can be ignored in our systems:

∆*E*_MM_ = ∆*E*_ele_ + ∆*E*_vdW_ + ∆*E*_int_. (3)

∆*G*_solv_ contains contributions from a polar part (∆*G*_GB_) and a non-polar (∆*G*_SA_) part:

∆*G*_solv_ = ∆*G*_GB_ + ∆*G*_SA_. (4)

∆*G*_GB_ was estimated by the generalized Born (GB) model with the interior and exterior dielectric constants set to 4 and 80, respectively [47,48]. The nonpolar solvation terms were calculated according to the LCPO algorithm:

∆*G*_SA_ = γ∆SASA + β, (5)

where SASA is solvent-accessible surface area, γ and β are set to 0.0072 kcal·mol^−1^·Å^−2^ and 0, respectively [49]. Therefore, the binding free energy was calculated as follows:

∆*G*_bind_ = ∆*E*_ele_ + ∆*E*_vdW_ + ∆*G*_GB_ + ∆*G*_SA_ − T∆*S.* (6)

Based on the extracted snapshots, the entropic contribution (T∆*S*) was evaluated through normal mode analysis (NMA) [27,50].

## 4. Conclusions

While G4 structures represent attractive drug targets, their highly charged backbones and lack of specific binding pockets leads to critical issues regarding binding specificity. In the current work, the selective binding mechanism of the *c-MYC* G4-specific cell penetrating thiazole peptide **TH3** was investigated through comparative studies with three other promotor G4s and with another thiazole peptide analogue. Our combined in-depth analyses revealed that in binding with the *c-MYC* G4, **TH3** can induce the formation of structure-specific sandwich-like frameworks with both the top and bottom G-tetrads and the corresponding 5′- and 3′-capping nucleotides, leading to its superior binding affinity relative to those of *c-KIT1*, *c-KIT2*, and BCL2 G4s. In addition, **TH3** showed promoted specificity for the *c-MYC* G4 relative to its analogue. Furthermore, adding a thiazole group through a peptide bond to **TH3** may extend π-π stacking interactions to the loop nucleotide dT_7_, and the dimethylamino groups of the 5′- and 3′-stacked **TH3** that orient in the same direction may be covalently connected with a proper linker, thus forming a *c-MYC* G4-specific clip. These modifications may increase the potency of **TH3** through increasing its binding affinity and specificity in combination with changing its binding stoichiometry. Overall, our study provides pivotal insights into the selective binding mechanism of thiazole peptide **TH3**, shedding new light on the design and development of drugs targeting the *c-MYC* G4 structure.

## Figures and Tables

**Table 1:** Molecular docking predicted interactions between neutral peptides **TH1**/**TH3** and the structures of *c-MYC*, *c-KIT1*, *c-KIT2*, and *BCL-2* G4s.

G4	Peptide ^a^	Hydrogen Bond	π-π Stacking	Affinity ^b^
*c-MYC*	**TH1**-5′	dG_2_@N2−H22···O2, dG_17_@N2−H22···N2,	dG_17_	−6.0
		dG_18_@N3···H1−N1		
	**TH1**-3′	dA_21_@N6−H62···O1, dG_15_@O3′···H1−N1	dG_10_, dG_15_	−7.4
	**TH3**-5′	dA_3_@N6−H61···O2, dG_13_@N2−H21···O2, dG_17_@N2−H22···N2, dG_18_@O4′···H1−N1	dG_2_, dG_13_, dG_17_	−7.5
	**TH3**-3′	dG_15_@N2−H22···N2, dT_20_@N3−H3···O2,	dG_6_, dG_10_, dG_15_	−8.0
		dA_21_@N6−H62···O1		
*c-KIT1*	**TH1**-5′	dG_9_@N2−H22···O2, dG_20_@N2−H22···N4 dG_19_@O6···H1−N1		−7.4
	**TH1**-3′	dG_19_@N2−H22···O1	dG_3_	−7.5
	**TH3**-5′	dG_9_@N2−H22···O3, dA_15_@N6−H62···N2, dG_16_@O6···H2−N1, dG_20_@N2−H22···O2, dG_21_@N2−H22···N4		−8.5
	**TH3**-3′	dG_2_@OP2···H2−N1, dG_3_@OP2···H3−N3,	dG_3_	−8.5
		dG_14_@N2−H22···O1, dG_19_@N2−H22···O3		
*c-KIT2*	**TH1**-5′	dG_6_@O4′···H2−N1, dA_12_@N6−H62···O2		−5.9
	**TH1**-3′	dG_20_@N1−H1···O1, dG_20_@N2−H21···O1	dG_3_	−5.6
	**TH3**-5′	dA_12_@N6−H61···N6, dA_12_@N6−H61···O3, dA_12_@N6−H62···N6	dG_1_, dG_5_	−6.7
	**TH3**-3′	dG_20_@O6···H4−N5, dG_20_@N1−H1···O1	dG_7_, dG_15_, dG_20_	−6.9
*BCL-2*	**TH1**	dG_5_@O3′···H2−N1, dG_5_@N2−H22···O1, dC_6_@N4−H42···N4	dG_5_, dC_6_	−6.3
	**TH3**	dG_5_@N2−H22···N2, dG_5_@O6···H4−N5, dC_6_@O4′···H2−N1, dG_23_@N2−H22···N6, dG_23_@N2−H22···O3	dG_5_	−6.8

^a^ 5′ and 3′ indicate the docking conformations of peptide **TH1**/**TH3** locate close to the 5′ and 3′ G-tetrads of G4s, respectively. ^b^ The energies are in kcal∙mol^−1^.

**Table 2:** Intermolecular hydrogen bonds formed between G4s and binding thiazole peptides ^1^.

Model	Acceptor	Donor		Ocpy. (%)	Dist. (Å)	Ang. (°)
*c-MYC* G4–**TH1**	**TH1**-5′@N2	dA_3_@H61	dA_3_@N6	36.67	3.11	151.48
	dA_3_@N1	**TH1**-5′@H1	**TH1**-5′@N1	24.79	3.02	162.95
	dA_3_@N1	**TH1**-5′@H2	**TH1**-5′@N1	15.81	3.01	163.01
	dT_20_@O4	**TH1**-3′@H1	**TH1**-3′@N1	45.50	2.93	161.67
	**TH1**-3′@N2	dA_21_@H62	dA_21_@N6	37.17	3.27	157.09
	dT_20_@O4	**TH1**-3′@H2	**TH1**-3′@N1	34.58	2.95	161.33
*c-MYC* G4–**TH3**	**TH3**-5′@O2	dA_3_@H61	dA_3_@N6	43.21	3.07	149.62
	**TH3**-3′@N2	dA_21_@H62	dA_21_@N6	32.24	3.27	157.13
	dT_7_@OP2	**TH3**-3′@H5	**TH3**-3′@N7	50.64	3.06	150.39
	dT_20_@O4	**TH3**-3′@H1	**TH3**-3′@N1	38.39	2.92	161.88
	dT_20_@O4	**TH3**-3′@H2	**TH3**-3′@N1	30.38	2.92	161.83
*c-KIT1* G4–**TH1**	dA_15_@O4′	**TH1**-3′@H1	**TH1**-3′@N1	35.48	3.00	155.56
	dA_18_@N1	**TH1**-3′@H2	**TH1**-3′@N1	34.73	3.05	157.50
*c-KIT1* G4–**TH3**	**TH3**-3′@O3	dG_19_@H22	dG_19_@N2	99.32	2.91	159.01
	**TH3**-3′@O1	dG_14_@H22	dG_14_@N2	99.12	2.92	153.79
	dG_3_@OP2	**TH3**-3′@H3	**TH3**-3′@N3	77.86	3.05	138.60
*c-KIT2* G4–**TH1**	dG_20_@O6	**TH1**-3′@H1	**TH1**-3′@N1	39.50	2.93	161.40
	dG_20_@O6	**TH1**-3′@H2	**TH1**-3′@N1	31.78	2.93	161.42
*c-KIT2* G4–**TH3**	**TH3**-5′@O2	dA_12_@H62	dA_12_@N6	57.36	2.96	153.41
	**TH3**-5′@O3	dA_12_@H61	dA_12_@N6	55.44	3.03	158.33
	dC_10_@O2	**TH3**-5′@H2	**TH3**-3′@N1	15.54	2.86	153.99
	dC_10_@O2	**TH3**-5′@H1	**TH3**-3′@N1	14.73	2.86	152.17
	dG_3_@OP2	**TH3**-3′@H5	**TH3**-3′@N7	34.43	3.02	153.99
*BCL2* G4–**TH1**	**TH1**@N1	dG_5_@H22	dG_5_@N2	45.99	3.09	156.96
	dC_6_@O5′	**TH1**@H3	**TH1**@N3	42.05	3.19	149.52
*BCL2* G4–**TH3**	dG_5_@OP2	**TH3**@H1	**TH3**@N1	17.04	2.91	159.19
	dG_5_@OP2	**TH3**@H2	**TH3**@N1	16.19	2.91	158.99

^1^ Ocpy., Dist., and Ang. are the occupancy, bond length, and bond angle of the Hoogsteen hydrogen bonds, respectively.

**Table 3:** Binding free energies between G4s and binding thiazole peptides.

G4	Peptide	Energy Components ^1^						
		Δ*E*_ele_	Δ*E*_vdW_	Δ*G*_GB_	Δ*G*_SA_	Δ*H*	−TΔ*S*	Δ*G*_bind_
*c-MYC*	**TH1**-5′	−2.9 ± 1.4	−39.0 ± 3.5	5.7 ± 1.1	−4.3 ± 0.3	−40.5 ± 3.5	20.0 ± 8.9	−20.5
	**TH1**-3′	−1.2 ± 1.6	−45.5 ± 3.7	4.9 ± 1.2	−5.4 ± 0.3	−47.2 ± 3.7	18.3 ± 9.0	−28.9
	**TH3**-5′	−2.5 ± 1.6	−53.9 ± 4.5	6.6 ± 1.4	−5.6 ± 0.3	−55.4 ± 4.3	20.5 ± 9.2	−34.9
	**TH3**-3′	−4.0 ± 2.0	−54.0 ± 4.2	7.8 ± 1.6	−6.3 ± 0.4	−56.5 ± 4.4	20.2 ± 9.0	−36.3
*c-KIT1*	**TH1**-5′	−1.9 ± 1.7	−32.3 ± 4.2	4.8 ± 1.6	−3.6 ± 0.4	−33.0 ± 4.2	17.2 ± 8.8	−15.8
	**TH1**-3′	−7.0 ± 1.1	−43.7 ± 3.7	9.4 ± 0.9	−4.4 ± 0.3	−45.8 ± 3.6	20.4 ± 9.3	−25.4
	**TH3**-5′	−1.4 ± 2.9	−42.4 ± 3.5	2.5 ± 2.4	−4.4 ± 0.4	−43.0 ± 3.6	21.0 ± 8.9	−22.0
	**TH3**-3′	−4.2 ± 2.0	−52.0 ± 3.2	7.5 ± 1.5	−5.8 ± 0.2	−54.5 ± 3.1	23.4 ± 8.9	−31.1
*c-KIT2*	**TH1**-5′	−2.0 ± 1.7	−28.6 ± 3.6	4.5 ± 1.4	−3.3 ± 0.4	−29.3 ± 3.6	18.4 ± 8.9	−10.9
	**TH1**-3′	−0.6 ± 1.4	−34.6 ± 2.8	3.3 ± 1.2	−4.1 ± 0.3	−35.9 ± 2.8	18.6 ± 9.1	−17.3
	**TH3**-5′	−3.2 ± 3.3	−41.7 ± 3.6	6.2 ± 3.0	−4.7 ± 0.4	−43.5 ± 4.0	21.1 ± 8.8	−22.4
	**TH3**-3′	−2.1 ± 2.9	−42.7 ± 6.2	6.5 ± 3.0	−4.7 ± 1.0	−43.1 ± 5.2	20.5 ± 9.4	−22.6
*BCL-2*	**TH1**	−9.0 ± 4.0	−22.4 ± 3.9	9.1 ± 3.3	−2.3 ± 0.5	−24.6 ± 4.5	17.7 ± 8.7	−6.9
	**TH3**	−1.8 ± 2.5	−25.6 ± 3.2	4.6 ± 2.2	−3.4 ± 0.4	−26.2 ± 3.4	19.1 ± 9.2	−7.1

^1^ Energies are in kcal∙mol^−1^.

## Data Availability

Data are contained within the article and Supplementary Materials.

## Supplementary Materials

The following supporting information can be downloaded at: https://www.mdpi.com/article/10.3390/ijms25010623/s1.
