# Supplementary material for: Molecular Insights into the Specific Targeting of c-MYC G-Quadruplex by Thiazole Peptides
Source: Int J Mol Sci. 2024 Jan 3;25(1):623. doi: 10.3390/ijms25010623 (PMC10778990; doi:10.3390/ijms25010623)
Supplement: Supplementary file 1 [file ijms-25-00623-s001.zip › ijms-2793863-supplementary.pdf]

Molecular Insights into the Specific Targeting of *c-MYC* G-quadruplex by Thiazole Peptides

Sen Cao[1,#], Qian Su[1,#], Yonghao Chen[2], Menglu Wang[2], Yi Xu[2], Lihui Wang[1], Yanhua Lu[1], Jianfeng Li[1], Jun Liu[1], Xiaojing Hong[1], Hongyan Wang[1], Jun-Ping Liu[1,*], Zhiguo Wang[1,*]

[1] Institute of Ageing Research, School of Basic Medical Sciences, Hangzhou Normal University, Hangzhou 311121, China

[2] School of Basic Medical Sciences, Hangzhou Normal University, Hangzhou 311121, China

[#] Both authors contributed equally to this work.

[*] To whom correspondence should be addressed:

Jun-Ping Liu, jun-ping.liu@hznu.edu.cn

Zhiguo Wang, zhgwang@hznu.edu.cn

Supplementary tables

Table S1. Sequence of the promotor G4s and the effects of thiazole peptides to G4 stabilization and cell proliferation[a].

G4s	Sequence	ΔT_m (°C)[b]		IC_{50} (μM)	
		**TH1**	**TH3**	**TH1**	**TH3**
c-MYC	TGAGGGTGGGTAGGGTGGGTAA	2.5	22.0	–[c]	3.8 ± 0.6[d]
c-KIT1	GGGAGGGCGCTGGGAGGAGGG	1.4	9.5	–	3.2 ± 0.4[e]
c-KIT2	GGGCGGGCGCTAGGGAGGGG	1.1	7.6		
BCL2	GGGCGCGGGAGGAATTGGGCGGG	2.0	7.1		

[a] The ΔT_m and IC_{50} data are retrieved from the reference 26. [b] ΔT_m are determined at 1 μM peptide concentration with a standard error of ± 1°C. [c] – means that data are not available. [d, e] IC_{50} values of **TH3** corresponding to the antiproliferation of HeLa and A549 cells, respectively.

Table S2. Molecular docking predicted interactions between peptides **TH1**/**TH3**[a] and the structures of *c-MYC*, *c-KIT1*, *c-KIT2*, and *BCL-2* G4s.

G4	Peptide[b]	Hydrogen bond	π-π stacking	Affinity[c]
c-MYC	**TH1**-5′	dG$_{2}$@N2−H22···O2, dG$_{17}$@O4′···H4−N5 dG$_{18}$@O4′···H2−N1	dG$_{17}$	−6.0
	**TH1**-3′	dA$_{21}$@N6−H61···O1	dG$_{10}$, dG$_{15}$	−6.7
	**TH3**-5′	dG$_{2}$@O6···H5−N7, dA$_{3}$@N6−H61···O2, dG$_{17}$@N2−H22···N2, dG$_{17}$@N3···H3−N3	dG$_{2}$, dG$_{13}$, dG$_{17}$	−7.5
	**TH3**-3′	dG$_{15}$@N2−H22···N2, dT$_{20}$@N3−H3···O2, dA$_{21}$@N6−H62···O1	dG$_{6}$, dG$_{10}$, dG$_{15}$	−8.5
c-KIT1	**TH1**-5′	dG$_{9}$@N2−H22···O2, dG$_{20}$@N2−H22···N4 dG$_{14}$@OP2···H2−N1		−7.0
	**TH1**-3′		dG$_{3}$	−6.5
	**TH3**-5′	dG$_{9}$@N2−H22···O3, dG$_{20}$@N2−H22···O2, dG$_{21}$@N2−H22···O1		−8.2
	**TH3**-3′	dG$_{2}$@OP2···H2−N1, dG$_{3}$@OP2···H3−N3, dG$_{14}$@N2−H22···O1, dG$_{19}$@N2−H22···O3	dG$_{3}$	−6.9
c-KIT2	**TH1**-5′	dG$_{6}$@O4′···H2−N1, dA$_{12}$@N6−H62···O2		−5.9
	**TH1**-3′	dG$_{20}$@N1−H1···O1, dG$_{20}$@N2−H21···O1	dG$_{3}$	−5.4
	**TH3**-5′	dA$_{12}$@N6−H61···N6, dA$_{12}$@N6−H62···O2 dA$_{12}$@N6−H61···O3	dG$_{1}$, dG$_{5}$	−7.1
	**TH3**-3′	dG$_{20}$@O6···H4−N5	dG$_{7}$, dG$_{15}$, dG$_{20}$	−6.7
BCL-2	**TH1**	dG$_{5}$@O3′···H2−N1, dG$_{5}$@N2−H22···O1, dC$_{6}$@N4−H42···N4	dG$_{5}$, dC$_{6}$	−6.2
	**TH3**	dG$_{5}$@N2−H22···N2, dG$_{5}$@N1−H1···O2, dG$_{23}$@N2−H22···O2	dG$_{5}$	−5.4

[a] The peptides are under the protonated state. [b] The words 5′ and 3′ indicate the docking conformations of peptide **TH1**/**TH3** locates close to the 5′ and 3′ G-tetrads of G4s, respectively. [c] The energies are in kcal·mol^{-1}.

Table S3. Hoogsteen hydrogen bonds of the apo and the peptides bound G4s.[1,2]

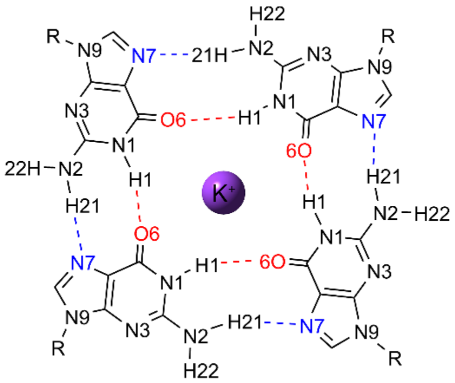

G4	Acceptor	Donor	Ocpy. (%)	Dist. (Å)	Ang. (°)
c-MYC	dG_{4}@O6	dG_{17}@H1 dG_{17}@N1	99.23%	2.93	155.26
	dG_{8}@O6	dG_{4}@H1 dG_{4}@N1	99.29%	2.98	160.52
	dG_{13}@O6	dG_{8}@H1 dG_{8}@N1	99.71%	2.92	158.36
	dG_{17}@O6	dG_{13}@H1 dG_{13}@N1	99.67%	2.94	158.37
	dG_{4}@N7	dG_{17}@H21 dG_{17}@N2	98.56%	3.04	158.08
	dG_{8}@N7	dG_{4}@H21 dG_{4}@N2	99.58%	2.98	159.01
	dG_{13}@N7	dG_{8}@H21 dG_{8}@N2	99.30%	3.00	159.33
	dG_{17}@N7	dG_{13}@H21 dG_{13}@N2	99.72%	2.97	160.09
	dG_{5}@O6	dG_{18}@H1 dG_{18}@N1	89.97%	3.17	138.95
	dG_{9}@O6	dG_{5}@H1 dG_{5}@N1	91.91%	3.19	141.05
	dG_{14}@O6	dG_{9}@H1 dG_{9}@N1	90.04%	3.19	139.53
	dG_{18}@O6	dG_{14}@H1 dG_{14}@N1	94.25%	3.17	141.01
	dG_{5}@N7	dG_{18}@H21 dG_{18}@N2	99.42%	2.96	158.53
	dG_{9}@N7	dG_{5}@H21 dG_{5}@N2	99.53%	2.99	157.49
	dG_{14}@N7	dG_{9}@H21 dG_{9}@N2	99.48%	2.98	157.52
	dG_{18}@N7	dG_{14}@H21 dG_{14}@N2	99.51%	3.00	156.55
	dG_{6}@O6	dG_{19}@H1 dG_{19}@N1	99.64%	2.95	158.25
	dG_{10}@O6	dG_{6}@H1 dG_{6}@N1	99.78%	2.97	158.98
	dG_{15}@O6	dG_{10}@H1 dG_{10}@N1	99.75%	2.94	159.30
	dG_{19}@O6	dG_{15}@H1 dG_{15}@N1	99.61%	2.98	161.50
	dG_{6}@N7	dG_{19}@H21 dG_{19}@N2	99.59%	2.96	157.03
	dG_{10}@N7	dG_{6}@H21 dG_{6}@N2	99.89%	2.97	161.24
	dG_{15}@N7	dG_{10}@H21 dG_{10}@N2	99.24%	2.99	155.25
	dG_{19}@N7	dG_{15}@H21 dG_{15}@N2	99.92%	2.96	158.96
c-MYC (**TH1**)	dG_{4}@O6	dG_{17}@H1 dG_{17}@N1	99.79%	2.94	159.07
	dG_{8}@O6	dG_{4}@H1 dG_{4}@N1	99.76%	2.94	159.53
	dG_{13}@O6	dG_{8}@H1 dG_{8}@N1	99.86%	2.93	159.62
	dG_{17}@O6	dG_{13}@H1 dG_{13}@N1	99.86%	2.93	159.63
	dG_{4}@N7	dG_{17}@H21 dG_{17}@N2	99.70%	2.99	159.48
	dG_{8}@N7	dG_{4}@H21 dG_{4}@N2	99.64%	2.98	159.16
	dG_{13}@N7	dG_{8}@H21 dG_{8}@N2	99.70%	2.99	160.78

	dG_{17}@N7	dG_{13}@H21 dG_{13}@N2	99.83%	2.98	160.00
	dG_{5}@O6	dG_{18}@H1 dG_{18}@N1	95.69%	3.12	142.71
	dG_{9}@O6	dG_{5}@H1 dG_{5}@N1	95.48%	3.14	143.38
	dG_{14}@O6	dG_{9}@H1 dG_{9}@N1	96.02%	3.12	143.93
	dG_{18}@O6	dG_{14}@H1 dG_{14}@N1	95.64%	3.14	143.09
	dG_{5}@N7	dG_{18}@H21 dG_{18}@N2	99.79%	2.95	161.21
	dG_{9}@N7	dG_{5}@H21 dG_{5}@N2	99.67%	2.98	160.48
	dG_{14}@N7	dG_{9}@H21 dG_{9}@N2	99.76%	2.96	161.04
	dG_{18}@N7	dG_{14}@H21 dG_{14}@N2	99.77%	2.96	159.66
	dG_{6}@O6	dG_{19}@H1 dG_{19}@N1	99.58%	2.96	162.03
	dG_{10}@O6	dG_{6}@H1 dG_{6}@N1	99.77%	2.95	161.75
	dG_{15}@O6	dG_{10}@H1 dG_{10}@N1	99.90%	2.92	162.32
	dG_{19}@O6	dG_{15}@H1 dG_{15}@N1	99.05%	2.99	157.81
	dG_{6}@N7	dG_{19}@H21 dG_{19}@N2	98.45%	3.02	152.95
	dG_{10}@N7	dG_{6}@H21 dG_{6}@N2	99.58%	3.00	157.96
	dG_{15}@N7	dG_{10}@H21 dG_{10}@N2	99.49%	3.00	156.51
	dG_{19}@N7	dG_{15}@H21 dG_{15}@N2	99.45%	3.01	156.47
c-MYC (**TH3**)	dG_{4}@O6	dG_{17}@H1 dG_{17}@N1	99.87%	2.92	158.85
	dG_{8}@O6	dG_{4}@H1 dG_{4}@N1	99.84%	2.92	161.90
	dG_{13}@O6	dG_{8}@H1 dG_{8}@N1	99.92%	2.92	160.79
	dG_{17}@O6	dG_{13}@H1 dG_{13}@N1	99.92%	2.93	161.55
	dG_{4}@N7	dG_{17}@H21 dG_{17}@N2	99.47%	3.01	157.05
	dG_{8}@N7	dG_{4}@H21 dG_{4}@N2	99.33%	3.01	156.87
	dG_{13}@N7	dG_{8}@H21 dG_{8}@N2	99.79%	2.98	158.05
	dG_{17}@N7	dG_{13}@H21 dG_{13}@N2	99.87%	2.97	159.84
	dG_{5}@O6	dG_{18}@H1 dG_{18}@N1	95.09%	3.13	141.84
	dG_{9}@O6	dG_{5}@H1 dG_{5}@N1	94.55%	3.16	142.59
	dG_{14}@O6	dG_{9}@H1 dG_{9}@N1	95.55%	3.14	143.08
	dG_{18}@O6	dG_{14}@H1 dG_{14}@N1	94.38%	3.16	141.68
	dG_{5}@N7	dG_{18}@H21 dG_{18}@N2	99.69%	2.96	161.20
	dG_{9}@N7	dG_{5}@H21 dG_{5}@N2	99.82%	2.98	160.63
	dG_{14}@N7	dG_{9}@H21 dG_{9}@N2	99.55%	2.98	157.84
	dG_{18}@N7	dG_{14}@H21 dG_{14}@N2	99.52%	2.97	157.08
	dG_{6}@O6	dG_{19}@H1 dG_{19}@N1	99.37%	2.98	159.53
	dG_{10}@O6	dG_{6}@H1 dG_{6}@N1	99.91%	2.93	160.87
	dG_{15}@O6	dG_{10}@H1 dG_{10}@N1	99.96%	2.91	162.87
	dG_{19}@O6	dG_{15}@H1 dG_{15}@N1	99.37%	2.99	160.53
	dG_{6}@N7	dG_{19}@H21 dG_{19}@N2	99.38%	2.98	155.92
	dG_{10}@N7	dG_{6}@H21 dG_{6}@N2	99.68%	3.00	158.54
	dG_{15}@N7	dG_{10}@H21 dG_{10}@N2	99.75%	3.00	156.65
	dG_{19}@N7	dG_{15}@H21 dG_{15}@N2	99.73%	2.98	155.76
c-KIT1	dG_{1}@O6	dG_{12}@H1 dG_{12}@N1	98.61%	2.96	153.94

	dG_5@O6	dG_1@H1 dG_1@N1	99.15%	2.97	157.35
	dG_9@O6	dG_5@H1 dG_5@N1	99.42%	2.94	156.91
	dG_{12}@O6	dG_9@H1 dG_9@N1	99.59%	2.94	161.03
	dG_1@N7	dG_{12}@H21 dG_{12}@N2	98.93%	3.00	158.84
	dG_5@N7	dG_1@H21 dG_1@N2	99.10%	2.99	158.79
	dG_9@N7	dG_5@H21 dG_5@N2	98.62%	3.03	160.29
	dG_{12}@N7	dG_9@H21 dG_9@N2	99.25%	3.01	159.41
	dG_2@O6	dG_{13}@H1 dG_{13}@N1	95.67%	3.11	142.44
	dG_6@O6	dG_2@H1 dG_2@N1	97.11%	3.11	144.74
	dG_{13}@O6	dG_{20}@H1 dG_{20}@N1	96.34%	3.12	144.18
	dG_{20}@O6	dG_6@H1 dG_6@N1	96.68%	3.12	144.75
	dG_2@N7	dG_{13}@H21 dG_{13}@N2	99.58%	2.97	161.79
	dG_6@N7	dG_2@H21 dG_2@N2	99.79%	2.96	162.51
	dG_{13}@N7	dG_{20}@H21 dG_{20}@N2	99.73%	2.97	163.30
	dG_{20}@N7	dG_6@H21 dG_6@N2	99.88%	2.95	162.62
	dG_3@O6	dG_{14}@H1 dG_{14}@N1	99.44%	2.99	156.30
	dG_7@O6	dG_3@H1 dG_3@N1	99.30%	2.98	156.69
	dG_{14}@O6	dG_{21}@H1 dG_{21}@N1	99.68%	2.98	158.08
	dG_{21}@O6	dG_7@H1 dG_7@N1	99.36%	2.98	155.34
	dG_3@N7	dG_{14}@H21 dG_{14}@N2	99.77%	2.96	160.53
	dG_7@N7	dG_3@H21 dG_3@N2	99.70%	2.99	161.74
	dG_{14}@N7	dG_{21}@H21 dG_{21}@N2	99.80%	2.99	161.17
	dG_{21}@N7	dG_7@H21 dG_7@N2	99.65%	3.00	160.02
c-KIT1 (**TH1**)	dG_1@O6	dG_{12}@H1 dG_{12}@N1	98.59%	2.97	151.97
	dG_5@O6	dG_1@H1 dG_1@N1	99.42%	2.98	155.92
	dG_9@O6	dG_5@H1 dG_5@N1	99.57%	2.96	157.74
	dG_{12}@O6	dG_9@H1 dG_9@N1	99.24%	2.97	158.23
	dG_1@N7	dG_{12}@H21 dG_{12}@N2	99.42%	2.98	159.85
	dG_5@N7	dG_1@H21 dG_1@N2	99.84%	2.97	162.11
	dG_9@N7	dG_5@H21 dG_5@N2	99.46%	3.00	162.04
	dG_{12}@N7	dG_9@H21 dG_9@N2	99.60%	2.99	161.73
	dG_2@O6	dG_{13}@H1 dG_{13}@N1	97.10%	3.11	144.37
	dG_6@O6	dG_2@H1 dG_2@N1	97.38%	3.11	144.84
	dG_{13}@O6	dG_{20}@H1 dG_{20}@N1	97.75%	3.10	145.24
	dG_{20}@O6	dG_6@H1 dG_6@N1	97.32%	3.11	145.04
	dG_2@N7	dG_{13}@H21 dG_{13}@N2	99.85%	2.95	162.93
	dG_6@N7	dG_2@H21 dG_2@N2	99.88%	2.96	163.15
	dG_{13}@N7	dG_{20}@H21 dG_{20}@N2	99.76%	2.98	164.01
	dG_{20}@N7	dG_6@H21 dG_6@N2	99.89%	2.95	163.39
	dG_3@O6	dG_{14}@H1 dG_{14}@N1	99.61%	2.98	157.65
	dG_7@O6	dG_3@H1 dG_3@N1	97.96%	3.00	156.42
	dG_{14}@O6	dG_{21}@H1 dG_{21}@N1	99.49%	2.98	156.45
	dG_{21}@O6	dG_7@H1 dG_7@N1	99.38%	2.98	156.02

	dG_3@N7	dG_{14}@H21 dG_{14}@N2	99.77%	2.96	161.66
	dG_7@N7	dG_3@H21 dG_3@N2	99.84%	2.98	162.00
	dG_{14}@N7	dG_{21}@H21 dG_{21}@N2	99.80%	2.98	161.02
	dG_{21}@N7	dG_7@H21 dG_7@N2	99.79%	3.00	162.27
c-KIT1 (**TH3**)	dG_1@O6	dG_{12}@H1 dG_{12}@N1	99.59%	2.92	158.85
	dG_5@O6	dG_1@H1 dG_1@N1	99.30%	2.96	157.70
	dG_9@O6	dG_5@H1 dG_5@N1	99.44%	2.94	154.52
	dG_{12}@O6	dG_9@H1 dG_9@N1	99.54%	2.93	161.54
	dG_1@N7	dG_{12}@H21 dG_{12}@N2	98.99%	3.02	158.20
	dG_5@N7	dG_1@H21 dG_1@N2	99.70%	2.96	158.60
	dG_9@N7	dG_5@H21 dG_5@N2	99.44%	3.00	160.18
	dG_{12}@N7	dG_9@H21 dG_9@N2	99.44%	3.00	160.34
	dG_2@O6	dG_{13}@H1 dG_{13}@N1	97.82%	3.09	146.78
	dG_6@O6	dG_2@H1 dG_2@N1	98.71%	3.07	144.64
	dG_{13}@O6	dG_{20}@H1 dG_{20}@N1	98.13%	3.06	146.42
	dG_{20}@O6	dG_6@H1 dG_6@N1	96.61%	3.08	143.46
	dG_2@N7	dG_{13}@H21 dG_{13}@N2	99.85%	2.91	161.39
	dG_6@N7	dG_2@H21 dG_2@N2	99.50%	2.96	157.70
	dG_{13}@N7	dG_{20}@H21 dG_{20}@N2	99.82%	2.97	164.07
	dG_{20}@N7	dG_6@H21 dG_6@N2	99.85%	2.94	160.96
	dG_3@O6	dG_{14}@H1 dG_{14}@N1	99.56%	2.97	161.13
	dG_7@O6	dG_3@H1 dG_3@N1	99.94%	2.94	163.63
	dG_{14}@O6	dG_{21}@H1 dG_{21}@N1	99.97%	2.86	162.90
	dG_{21}@O6	dG_7@H1 dG_7@N1	99.96%	2.90	165.45
	dG_3@N7	dG_{14}@H21 dG_{14}@N2	98.79%	3.04	148.14
	dG_7@N7	dG_3@H21 dG_3@N2	99.56%	3.03	157.43
	dG_{14}@N7	dG_{21}@H21 dG_{21}@N2	99.77%	2.97	154.23
	dG_{21}@N7	dG_7@H21 dG_7@N2	94.95%	3.12	151.15
c-KIT2	dG_1@O6	dG_{17}@H1 dG_{17}@N1	99.33%	2.94	156.72
	dG_5@O6	dG_1@H1 dG_1@N1	99.67%	2.95	158.79
	dG_{13}@O6	dG_5@H1 dG_5@N1	99.85%	2.91	159.92
	dG_{17}@O6	dG_{13}@H1 dG_{13}@N1	99.60%	2.94	161.07
	dG_1@N7	dG_{17}@H21 dG_{17}@N2	99.22%	3.01	159.15
	dG_5@N7	dG_1@H21 dG_1@N2	99.19%	3.00	157.78
	dG_{13}@N7	dG_5@H21 dG_5@N2	99.58%	3.00	160.38
	dG_{17}@N7	dG_{13}@H21 dG_{13}@N2	99.29%	2.99	157.67
	dG_2@O6	dG_{18}@H1 dG_{18}@N1	94.55%	3.13	141.19
	dG_6@O6	dG_2@H1 dG_2@N1	93.57%	3.17	141.99
	dG_{14}@O6	dG_6@H1 dG_6@N1	94.92%	3.14	142.37
	dG_{18}@O6	dG_{14}@H1 dG_{14}@N1	93.16%	3.18	141.42
	dG_2@N7	dG_{18}@H21 dG_{18}@N2	99.62%	2.96	160.26
	dG_6@N7	dG_2@H21 dG_2@N2	99.53%	2.98	158.85

	dG_{14}@N7	dG_{6}@H21 dG_{6}@N2	99.79%	2.95	160.88
	dG_{18}@N7	dG_{14}@H21 dG_{14}@N2	99.53%	3.00	158.01
	dG_{3}@O6	dG_{19}@H1 dG_{19}@N1	99.47%	2.97	158.72
	dG_{7}@O6	dG_{3}@H1 dG_{3}@N1	99.61%	2.98	159.30
	dG_{15}@O6	dG_{7}@H1 dG_{7}@N1	99.64%	2.97	158.53
	dG_{19}@O6	dG_{15}@H1 dG_{15}@N1	99.83%	2.96	159.87
	dG_{3}@N7	dG_{19}@H21 dG_{19}@N2	99.41%	2.99	157.10
	dG_{7}@N7	dG_{3}@H21 dG_{3}@N2	99.76%	2.98	160.33
	dG_{15}@N7	dG_{7}@H21 dG_{7}@N2	99.61%	2.98	158.44
	dG_{19}@N7	dG_{15}@H21 dG_{15}@N2	99.90%	2.96	160.90
c-KIT2 (**TH1**)	dG_{1}@O6	dG_{17}@H1 dG_{17}@N1	99.60%	2.94	157.68
	dG_{5}@O6	dG_{1}@H1 dG_{1}@N1	99.26%	2.97	158.54
	dG_{13}@O6	dG_{5}@H1 dG_{5}@N1	99.45%	2.93	156.49
	dG_{17}@O6	dG_{13}@H1 dG_{13}@N1	99.47%	2.96	158.36
	dG_{1}@N7	dG_{17}@H21 dG_{17}@N2	99.51%	3.00	160.91
	dG_{5}@N7	dG_{1}@H21 dG_{1}@N2	99.44%	2.98	159.88
	dG_{13}@N7	dG_{5}@H21 dG_{5}@N2	99.55%	3.00	161.05
	dG_{17}@N7	dG_{13}@H21 dG_{13}@N2	99.40%	2.97	158.83
	dG_{2}@O6	dG_{18}@H1 dG_{18}@N1	95.21%	3.14	142.87
	dG_{6}@O6	dG_{2}@H1 dG_{2}@N1	93.84%	3.17	141.85
	dG_{14}@O6	dG_{6}@H1 dG_{6}@N1	94.91%	3.13	141.55
	dG_{18}@O6	dG_{14}@H1 dG_{14}@N1	94.77%	3.15	142.71
	dG_{2}@N7	dG_{18}@H21 dG_{18}@N2	99.80%	2.95	161.29
	dG_{6}@N7	dG_{2}@H21 dG_{2}@N2	99.67%	2.97	158.32
	dG_{14}@N7	dG_{6}@H21 dG_{6}@N2	99.67%	2.97	160.36
	dG_{18}@N7	dG_{14}@H21 dG_{14}@N2	99.63%	2.98	159.84
	dG_{3}@O6	dG_{19}@H1 dG_{19}@N1	99.65%	2.97	159.65
	dG_{7}@O6	dG_{3}@H1 dG_{3}@N1	99.80%	2.95	160.70
	dG_{15}@O6	dG_{7}@H1 dG_{7}@N1	99.64%	2.96	159.65
	dG_{19}@O6	dG_{15}@H1 dG_{15}@N1	99.60%	2.96	158.03
	dG_{3}@N7	dG_{19}@H21 dG_{19}@N2	99.50%	2.99	157.43
	dG_{7}@N7	dG_{3}@H21 dG_{3}@N2	99.80%	2.98	159.07
	dG_{15}@N7	dG_{7}@H21 dG_{7}@N2	99.75%	2.98	158.93
	dG_{19}@N7	dG_{15}@H21 dG_{15}@N2	99.76%	2.99	159.04
c-KIT2 (**TH3**)	dG_{1}@O6	dG_{17}@H1 dG_{17}@N1	99.75%	2.94	158.59
	dG_{5}@O6	dG_{1}@H1 dG_{1}@N1	99.36%	2.95	159.97
	dG_{13}@O6	dG_{5}@H1 dG_{5}@N1	99.43%	2.92	154.02
	dG_{17}@O6	dG_{13}@H1 dG_{13}@N1	99.73%	2.95	161.15
	dG_{1}@N7	dG_{17}@H21 dG_{17}@N2	99.50%	3.00	159.45
	dG_{5}@N7	dG_{1}@H21 dG_{1}@N2	99.88%	2.97	160.49
	dG_{13}@N7	dG_{5}@H21 dG_{5}@N2	99.12%	3.02	159.90
	dG_{17}@N7	dG_{13}@H21 dG_{13}@N2	99.57%	2.98	158.22
	dG_{2}@O6	dG_{18}@H1 dG_{18}@N1	95.65%	3.13	142.67

	dG_6@O6	dG_2@H1 dG_2@N1	94.59%	3.16	142.42
	dG_{14}@O6	dG_6@H1 dG_6@N1	94.43%	3.13	141.22
	dG_{18}@O6	dG_{14}@H1 dG_{14}@N1	95.16%	3.15	142.84
	dG_2@N7	dG_{18}@H21 dG_{18}@N2	99.75%	2.94	160.40
	dG_6@N7	dG_2@H21 dG_2@N2	99.75%	2.96	159.23
	dG_{14}@N7	dG_6@H21 dG_6@N2	99.69%	2.97	160.42
	dG_{18}@N7	dG_{14}@H21 dG_{14}@N2	99.70%	2.98	159.86
	dG_3@O6	dG_{19}@H1 dG_{19}@N1	99.78%	2.95	159.91
	dG_7@O6	dG_3@H1 dG_3@N1	99.75%	2.97	160.58
	dG_{15}@O6	dG_7@H1 dG_7@N1	99.54%	2.97	158.15
	dG_{19}@O6	dG_{15}@H1 dG_{15}@N1	99.88%	2.95	159.95
	dG_3@N7	dG_{19}@H21 dG_{19}@N2	99.64%	2.99	158.48
	dG_7@N7	dG_3@H21 dG_3@N2	99.82%	2.98	159.80
	dG_{15}@N7	dG_7@H21 dG_7@N2	99.55%	2.98	157.90
	dG_{19}@N7	dG_{15}@H21 dG_{15}@N2	99.90%	2.97	160.55
BCL-2	dG_1@O6	dG_9@H1 dG_9@N1	99.96%	2.93	161.16
	dG_9@O6	dG_{17}@H1 dG_{17}@N1	99.91%	2.93	162.12
	dG_{17}@O6	dG_{21}@H1 dG_{21}@N1	99.93%	2.93	159.65
	dG_{21}@O6	dG_1@H1 dG_1@N1	99.82%	2.92	159.16
	dG_1@N7	dG_9@H21 dG_9@N2	99.94%	2.96	160.22
	dG_9@N7	dG_{17}@H21 dG_{17}@N2	99.69%	3.00	154.48
	dG_{17}@N7	dG_{21}@H21 dG_{21}@N2	99.63%	3.00	160.74
	dG_{21}@N7	dG_1@H21 dG_1@N2	99.44%	3.04	155.38
	dG_2@O6	dG_{22}@H1 dG_{22}@N1	91.87%	3.18	141.17
	dG_8@O6	dG_2@H1 dG_2@N1	87.10%	3.22	140.12
	dG_{18}@O6	dG_8@H1 dG_8@N1	94.12%	3.14	142.46
	dG_{22}@O6	dG_{18}@H1 dG_{18}@N1	92.35%	3.18	141.19
	dG_2@N7	dG_{22}@H21 dG_{22}@N2	99.74%	2.98	158.56
	dG_8@N7	dG_2@H21 dG_2@N2	99.01%	3.03	160.84
	dG_{18}@N7	dG_8@H21 dG_8@N2	99.68%	2.99	162.50
	dG_{22}@N7	dG_{18}@H21 dG_{18}@N2	99.84%	2.96	160.50
	dG_3@O6	dG_{23}@H1 dG_{23}@N1	99.86%	2.97	161.57
	dG_7@O6	dG_3@H1 dG_3@N1	98.98%	3.00	154.06
	dG_{19}@O6	dG_7@H1 dG_7@N1	99.97%	2.89	159.63
	dG_{23}@O6	dG_{19}@H1 dG_{19}@N1	99.21%	2.99	152.93
	dG_3@N7	dG_{23}@H21 dG_{23}@N2	99.90%	2.96	158.88
	dG_7@N7	dG_3@H21 dG_3@N2	99.93%	2.96	162.45
	dG_{19}@N7	dG_7@H21 dG_7@N2	99.96%	2.96	160.15
	dG_{23}@N7	dG_{19}@H21 dG_{19}@N2	99.49%	3.00	160.56
BCL-2 **(TH1)**	dG_1@O6	dG_9@H1 dG_9@N1	99.97%	2.92	161.20
	dG_9@O6	dG_{17}@H1 dG_{17}@N1	99.91%	2.92	162.15
	dG_{17}@O6	dG_{21}@H1 dG_{21}@N1	99.92%	2.93	159.31

	dG_{21}@O6	dG_{1}@H1 dG_{1}@N1	99.82%	2.92	159.11
	dG_{1}@N7	dG_{9}@H21 dG_{9}@N2	99.94%	2.95	160.30
	dG_{9}@N7	dG_{17}@H21 dG_{17}@N2	99.68%	3.00	154.20
	dG_{17}@N7	dG_{21}@H21 dG_{21}@N2	99.56%	3.01	160.54
	dG_{21}@N7	dG_{1}@H21 dG_{1}@N2	99.46%	3.04	155.38
	dG_{2}@O6	dG_{22}@H1 dG_{22}@N1	91.81%	3.18	140.78
	dG_{8}@O6	dG_{2}@H1 dG_{2}@N1	87.78%	3.22	140.37
	dG_{18}@O6	dG_{8}@H1 dG_{8}@N1	93.34%	3.16	142.18
	dG_{22}@O6	dG_{18}@H1 dG_{18}@N1	92.25%	3.19	141.18
	dG_{2}@N7	dG_{22}@H21 dG_{22}@N2	99.70%	2.98	158.11
	dG_{8}@N7	dG_{2}@H21 dG_{2}@N2	99.04%	3.03	161.21
	dG_{18}@N7	dG_{8}@H21 dG_{8}@N2	99.69%	2.99	162.21
	dG_{22}@N7	dG_{18}@H21 dG_{18}@N2	99.82%	2.96	160.28
	dG_{3}@O6	dG_{23}@H1 dG_{23}@N1	99.92%	2.95	161.06
	dG_{7}@O6	dG_{3}@H1 dG_{3}@N1	99.57%	2.97	155.82
	dG_{19}@O6	dG_{7}@H1 dG_{7}@N1	99.96%	2.91	160.96
	dG_{23}@O6	dG_{19}@H1 dG_{19}@N1	99.32%	3.00	154.05
	dG_{3}@N7	dG_{23}@H21 dG_{23}@N2	99.91%	2.97	159.43
	dG_{7}@N7	dG_{3}@H21 dG_{3}@N2	99.93%	2.96	162.08
	dG_{19}@N7	dG_{7}@H21 dG_{7}@N2	99.95%	2.97	159.76
	dG_{23}@N7	dG_{19}@H21 dG_{19}@N2	99.59%	3.00	161.14
BCL-2 (**TH3**)	dG_{1}@O6	dG_{9}@H1 dG_{9}@N1	99.97%	2.93	161.09
	dG_{9}@O6	dG_{17}@H1 dG_{17}@N1	99.90%	2.93	162.01
	dG_{17}@O6	dG_{21}@H1 dG_{21}@N1	99.94%	2.93	159.84
	dG_{21}@O6	dG_{1}@H1 dG_{1}@N1	99.84%	2.92	158.85
	dG_{1}@N7	dG_{9}@H21 dG_{9}@N2	99.95%	2.95	160.42
	dG_{9}@N7	dG_{17}@H21 dG_{17}@N2	99.69%	3.00	154.34
	dG_{17}@N7	dG_{21}@H21 dG_{21}@N2	99.75%	3.00	160.70
	dG_{21}@N7	dG_{1}@H21 dG_{1}@N2	99.36%	3.04	155.08
	dG_{2}@O6	dG_{22}@H1 dG_{22}@N1	92.86%	3.17	141.33
	dG_{8}@O6	dG_{2}@H1 dG_{2}@N1	88.24%	3.21	140.13
	dG_{18}@O6	dG_{8}@H1 dG_{8}@N1	94.65%	3.14	142.62
	dG_{22}@O6	dG_{18}@H1 dG_{18}@N1	93.12%	3.18	141.43
	dG_{2}@N7	dG_{22}@H21 dG_{22}@N2	99.80%	2.97	158.52
	dG_{8}@N7	dG_{2}@H21 dG_{2}@N2	99.18%	3.02	160.95
	dG_{18}@N7	dG_{8}@H21 dG_{8}@N2	99.73%	2.98	162.65
	dG_{22}@N7	dG_{18}@H21 dG_{18}@N2	99.86%	2.96	160.84
	dG_{3}@O6	dG_{23}@H1 dG_{23}@N1	99.88%	2.96	162.44
	dG_{7}@O6	dG_{3}@H1 dG_{3}@N1	99.10%	2.99	154.74
	dG_{19}@O6	dG_{7}@H1 dG_{7}@N1	99.97%	2.89	160.24
	dG_{23}@O6	dG_{19}@H1 dG_{19}@N1	99.40%	2.98	153.55
	dG_{3}@N7	dG_{23}@H21 dG_{23}@N2	99.91%	2.96	158.41

dG_7@N7	dG_3@H21	dG_3@N2	99.91%	2.97	161.56
dG_{19}@N7	dG_7@H21	dG_7@N2	99.95%	2.96	159.63
dG_{23}@N7	dG_{19}@H21	dG_{19}@N2	99.56%	3.00	160.31

[1] Hydrogen bonds located in the top, central, and bottom G-tetrads are colored in green, orange, and purple, respectively.

[2] Ocpy., Dist., and Ang. mean the occupancy, bond length, and bond angle of the Hoogsteen hydrogen bonds, respectively.

Supplementary figures

Figure S1

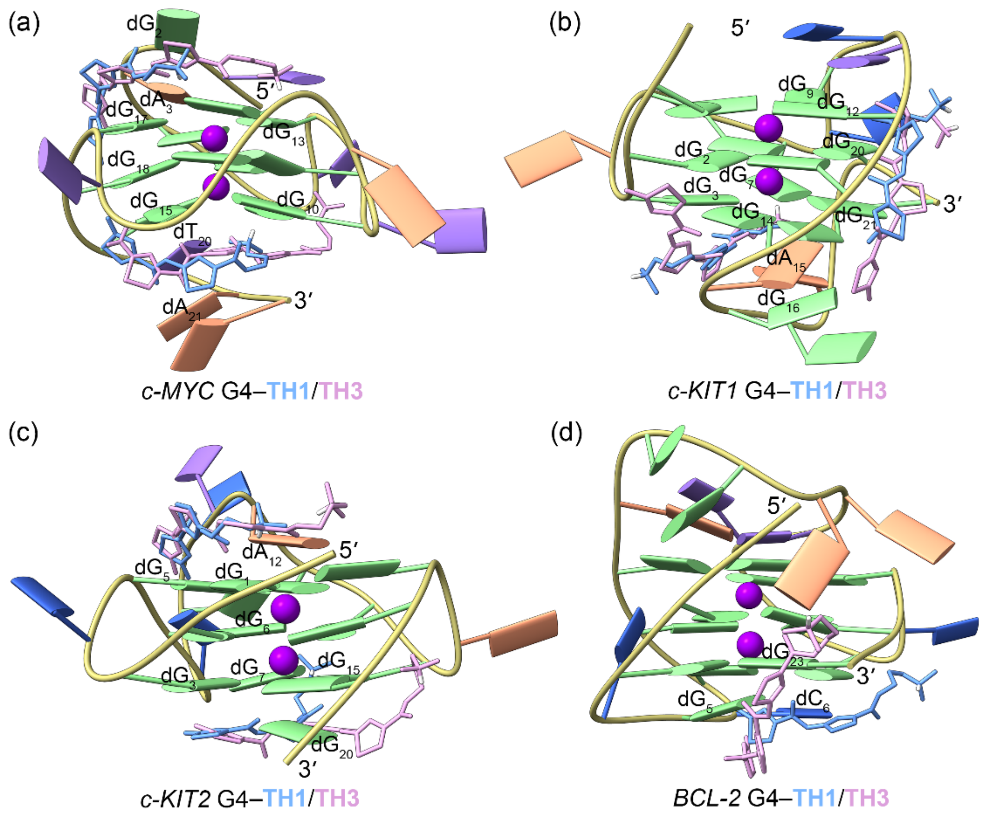

Figure S1. Binding modes of the **TH1**/**TH3** and the MD-equilibrated promotor G4s derived from molecular docking calculations. The **TH1** and **TH3** are under the protonated state and are colored cornflower blue and plum, respectively. The central potassium ions are represented by magenta spheres.

Figure S2

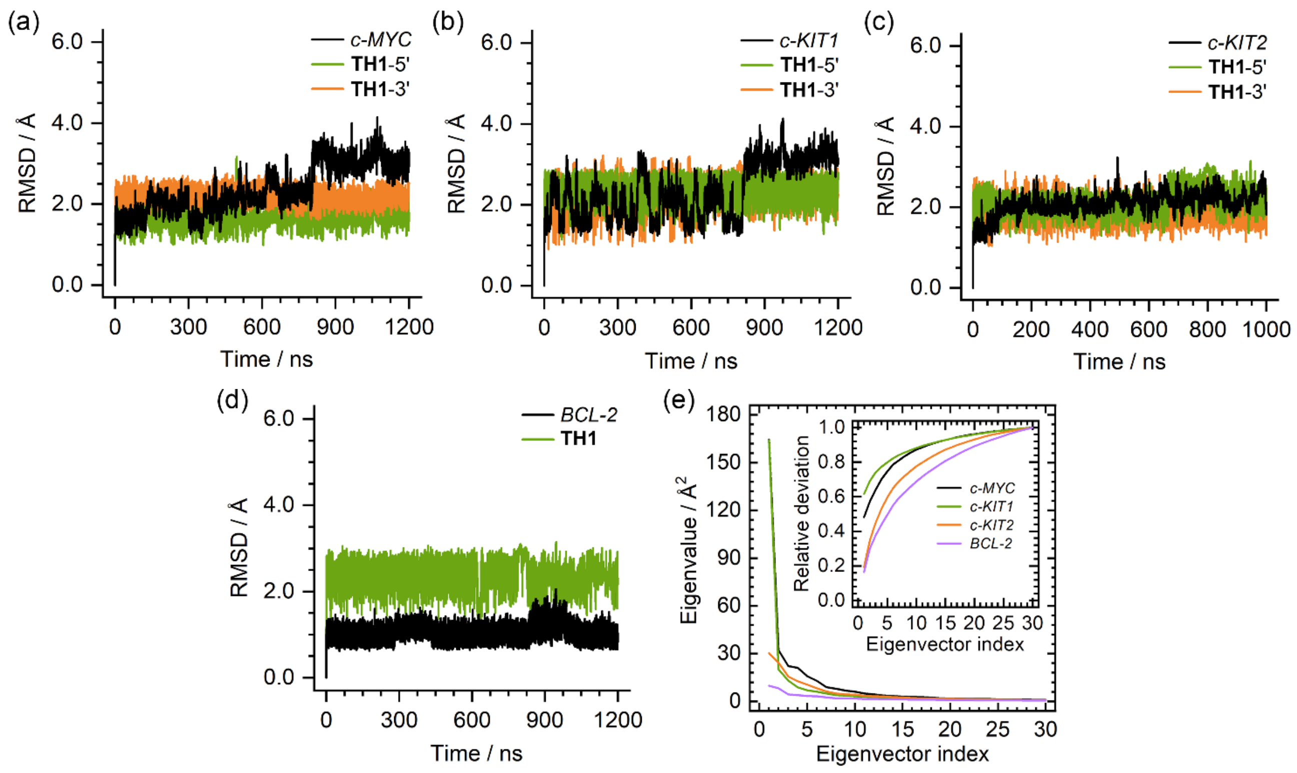

Figure S2. RMSD profiles of the **TH1** bound promotor G4s (a)−(d) and the eigenvalue profiles constructed by the first 30 eigenvectors of the **TH1** bound G4s (e).

Figure S3

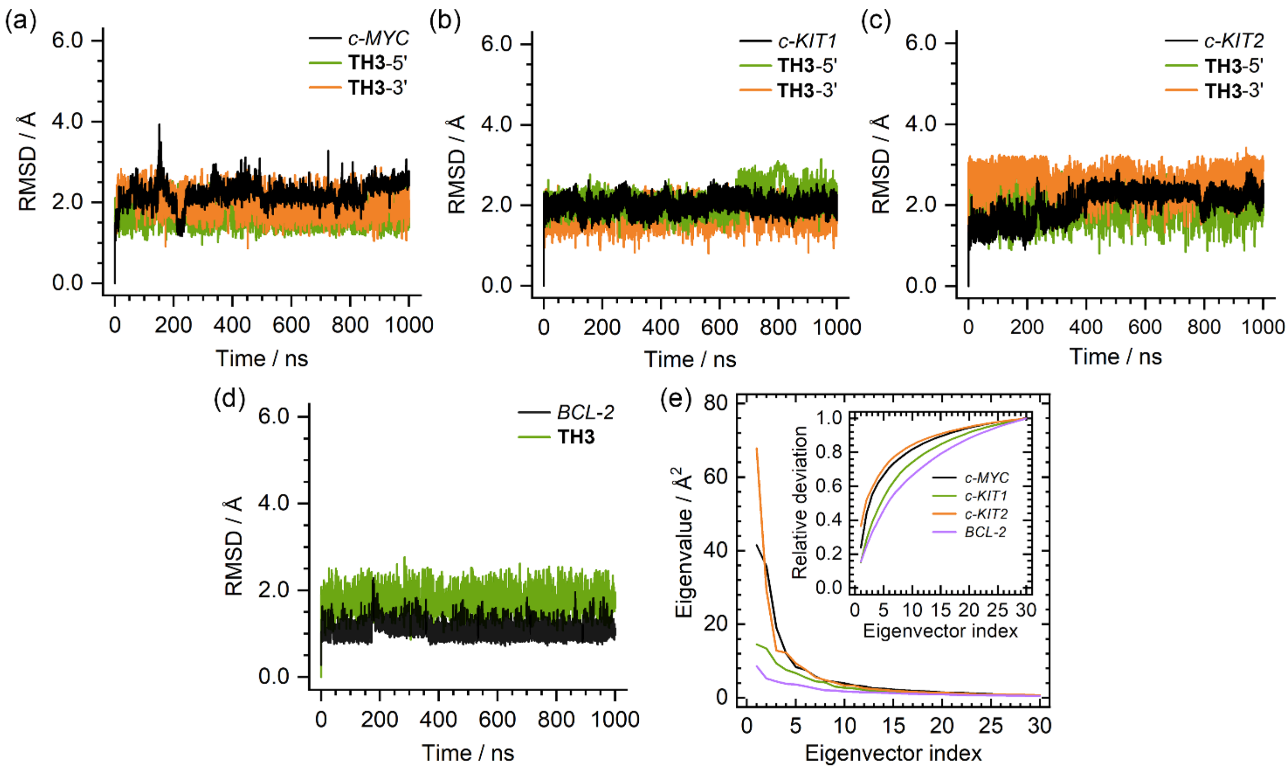

Figure S3. RMSD profiles of the **TH3** bound promotor G4s (a)−(d) and the eigenvalue profiles constructed by the first 30 eigenvectors of the **TH3** bound G4s (e).

Figure S4

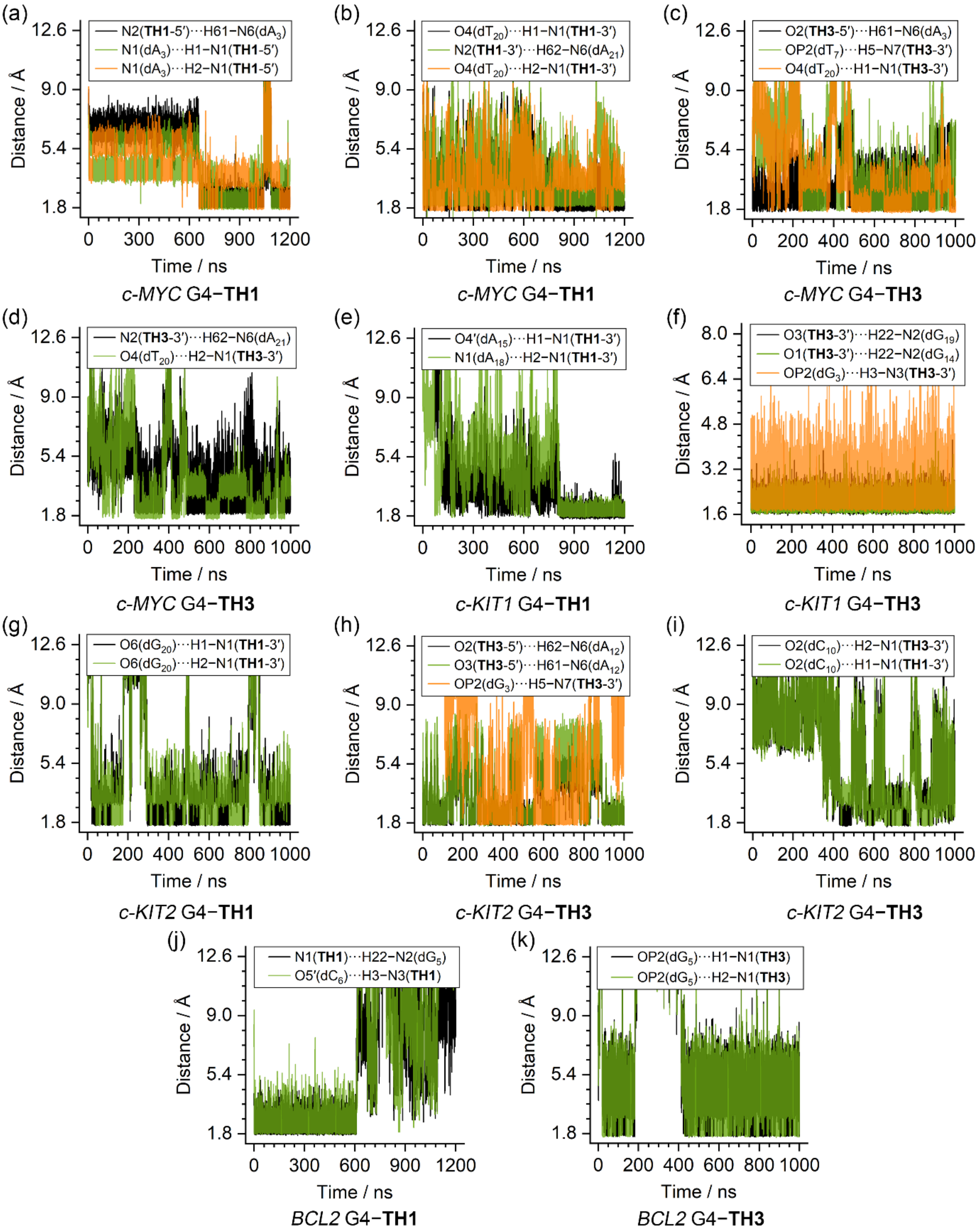

Figure S4. The variations of distance between the polar hydrogen and the hydrogen bond receptor atoms throughout the MD simulations.
